# The m6A demethylase ALKBH5 promotes tumor progression by inhibiting RIG-I expression and interferon alpha production through the IKKε/TBK1/IRF3 pathway in head and neck squamous cell carcinoma

**DOI:** 10.1186/s12943-022-01572-2

**Published:** 2022-04-09

**Authors:** Shufang Jin, Mingyu Li, Hanyue Chang, Ruijie Wang, Zhiyuan Zhang, Jianjun Zhang, Yue He, Hailong Ma

**Affiliations:** 1 Department of Oral Maxillofacial-Head and Neck Oncology, Shanghai Ninth People’s Hospital, College of Stomatology, Shanghai Jiao Tong University School of Medicine, No 639, Zhizaoju Rd, Shanghai, 200011 China; 2 National Clinical Research Center for Oral Diseases, Shanghai, 200011 China; 3 Shanghai Key Laboratory of Stomatology & Shanghai Research Institute of Stomatology, Shanghai, 200011 China; 4 Department of Second Dental Center, Shanghai Ninth People’s Hospital, Shanghai Jiao Tong University School of Medicine; College of Stomatology, Shanghai Jiao Tong University, Shanghai, 201900 China

**Keywords:** ALKBH5, N6-methyladenosine, RIG-I, Interferon alpha, Head and neck squamous cell carcinoma

## Abstract

**Background:**

N6-methyladenosine (m6A) RNA modification plays a critical role in various physiological and pathological conditions. However, the role of m6A modification in head and neck squamous cell carcinoma (HNSCC) remains elusive.

**Methods:**

In this study, the expression of m6A demethylases was detected by HNSCC tissue microarray. m6A-RNA immunoprecipitation (MeRIP) sequencing and RNA sequencing were used to identify downstream targets of ALKBH5. Comprehensive identification of RNA-binding proteins by mass spectrometry (ChIRP-MS) was used to explore the m6A “readers”. Tumor-infiltrating lymphocytes were analyzed in SCC7-bearing xenografts in C3H mice.

**Results:**

Here, we demonstrate the downregulation of m6A status and upregulation of two demethylases in HNSCC. Silencing the m6A demethylase alkB homolog 5, RNA demethylase (ALKBH5) suppresses tumor progression *in vitro* and *in vivo*. m6A-RNA immunoprecipitation sequencing reveals that ALKBH5 downregulates the m6A modification of *DDX58* mRNA. Moreover, RIG-I, encoded by the *DDX58* mRNA, reverses the protumorigenic characteristics of ALKBH5. ChIRP-MS demonstrates that HNRNPC binds to the m6A sites of *DDX58* mRNA to promote its maturation. ALKBH5 overexpression inhibits RIG-I-mediated IFNα secretion through the IKKε/TBK1/IRF3 pathway. The number of tumor-infiltrating lymphocytes in C3H immunocompetent mice is reduced by ALKBH5 overexpression and restored by IFNα administration. Upregulation of AKLBH5 negatively correlates with RIG-I and IFNα expression in HNSCC patients.

**Conclusions:**

These findings unveil a novel mechanism of immune microenvironment regulation mediated by m6A modification through the ALKBH5/RIG-I/IFNα axis, providing a rationale for therapeutically targeting epitranscriptomic modulators in HNSCC.

**Supplementary Information:**

The online version contains supplementary material available at 10.1186/s12943-022-01572-2.

## Background

N6-methyladenosine (m6A) modification is the most abundant of more than 100 modifications of eukaryotic mRNA [1]. It is a dynamic RNA modification that is added by “writers” methyltransferase-like 3 (METTL3), methyltransferase-like 14 (METTL14), and Wilms tumor-associated protein (WTAP) [2, 3], erased by “erasers” fat-mass and obesity-associated protein (FTO) [4] and α-ketoglutarate-dependent dioxygenase alkB homolog 5 (ALKBH5) [5], and recognized by “readers”, such as YTH domain family members (YTHDF1–3, YTHDC1/2), insulin growth factor-2 mRNA-binding proteins (IGF2BPs), and heterogeneous nuclear ribonucleoprotein (HNRNP) family members [6–8]. Accumulating evidence shows that m6A modification regulates the stability, localization, export, splicing, and translation of RNA at the posttranscriptional level [9] and thereby plays a critical role in cell reprogramming [10], spermatogenesis [11], T-cell homeostasis [12], and endothelial hematopoietic transformation [13]. Dysregulation of m6A modification is closely related to the occurrence and development of carcinomas such as glioblastoma, breast cancer, gastric cancer, and colorectal cancer [14–17]. However, the study of the m6A-mediated epitranscriptome has just begun, and the role of m6A modification in tumor progression needs further investigation.

Head and neck squamous cell carcinoma (HNSCC) seriously destroys chewing, breathing, swallowing, and other basic physiological functions and can even be life-threatening [18]. The 5-year survival rate of HNSCC is 50–60% after comprehensive treatment, including surgery, chemoradiotherapy, and targeted therapies [19]. It is urgent to explore the factors that drive tumor initiation and progression in HNSCC. The study of m6A modification opens a new perspective on posttranscriptional regulation. However, the modification status of m6A and how it participates in the progression of HNSCC are largely unknown.

Our previous studies focused mainly on the immune regulation and escape function of interferon alpha (IFNα) in HNSCC. IFNα is a pleiotropic cytokine that is produced by most nucleated cells and plays an indispensable role in immune cell differentiation and activation, antigen presentation, costimulatory mechanisms, and immune surveillance [20]. IFNα enhances the effect of targeted therapies by upregulating retinoic acid inducible gene I (RIG-I), which is a critical cytosolic pattern recognition receptor that is essential for detecting viral RNA and initiating the innate immune response [21]. However, the overactivation of IFNα signaling promotes the formation of an immunosuppressive microenvironment by upregulating programmed cell death ligand 1 (PD-L1) [22, 23]. Because IFNα acts as a double-edged sword in HNSCC treatment, it is very important to explore the upstream regulators of IFNα secretion in the tumor microenvironment.

In the present study, we sought to determine the status and underlying molecular mechanism of m6A modification during disease progression and IFNα production in HNSCC. We first demonstrated the function of ALKBH5 in facilitating HNSCC progression and the regulation of IFNα secretion to promote immune escape via m6A modification, which provides new insights into carcinogenesis and a novel potential target for cancer treatment.

## Results

### Downregulated m6A status and upregulated m6A demethylase expression are correlated with poor prognosis in HNSCC

**Fig. 1:**
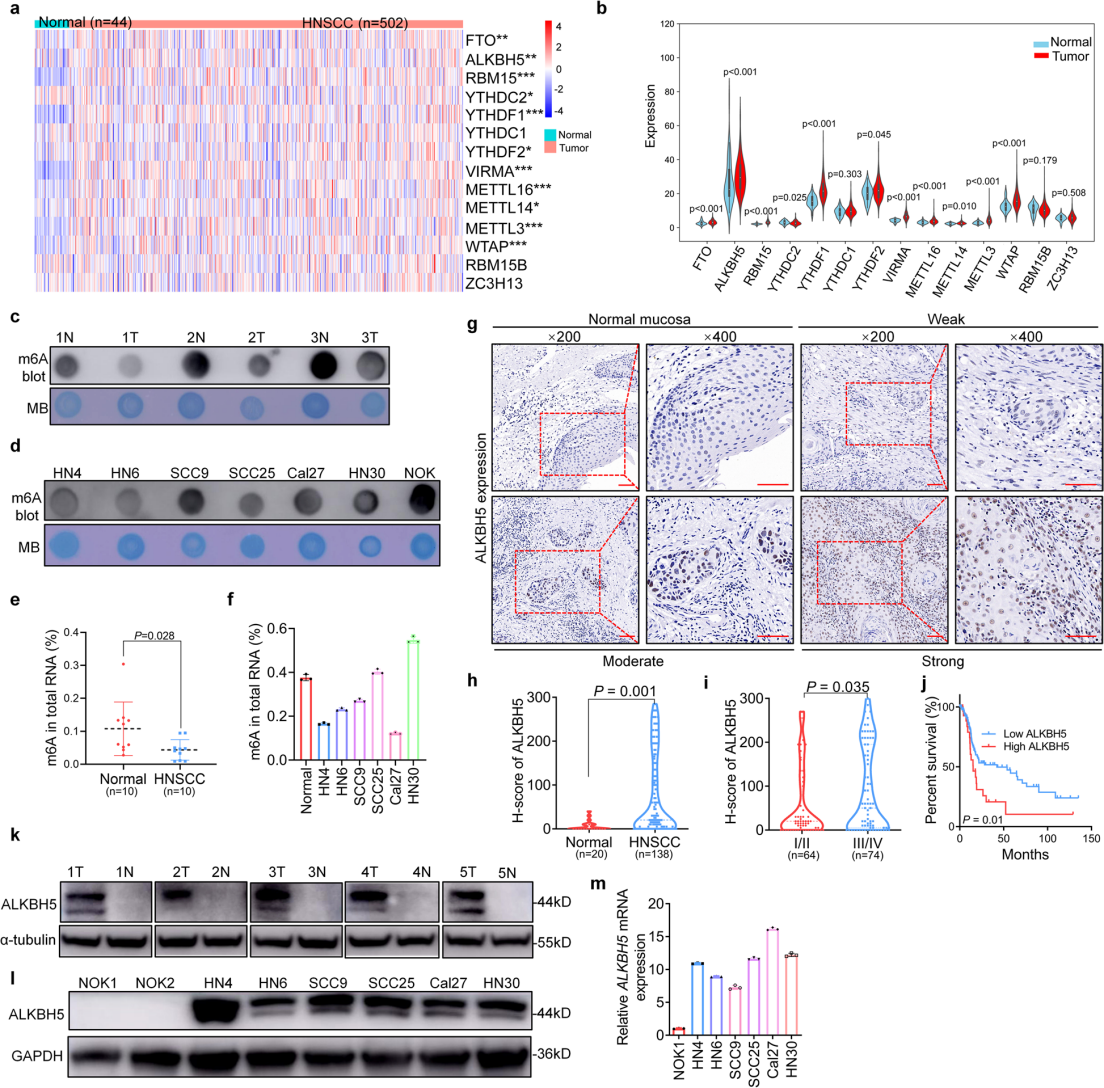
Downregulated m6A status and upregulated m6A demethylase are correlated with poor prognosis in HNSCC. **a**, **b** Heatmap profiling and violin plot analysis of the expression of m6A WERs in the TCGA HNSCC database. **c**, **d** Dot blotting using an m6A antibody was performed in paired HNSCC tissues and cell lines. **e**, **f** The percentage of m6A was detected in 10 paired HNSCC tissues and cell lines using an m6A RNA Methylation Quantification Kit. **g** Representative images of immunohistochemistry (IHC) staining for ALKBH5 on a tissue microarray (TMA) composed of 138 HNSCC tissues and 20 normal epithelium tissues. Scale bars: 100 μm. **h** The expression levels of ALKBH5 were analyzed in HNSCC and normal control tissues. **i** The correlation between TNM stage and ALKBH5 expression was analyzed. **j** The Kaplan–Meier method was used to plot survival curves for patients with high and low ALKBH5 expression in The Cancer Genome Atlas (TCGA) HNSCC dataset. The log-rank test was used to compare the survival rate. **k** Immunoblot analyses of five paired HNSCC tissues were performed with the indicated antibodies. **l** ALKBH5 expression was detected in six HNSCC cell lines and two normal oral keratinocyte (NOK) lines using immunoblotting. **m** ALKBH5 mRNA was analyzed by qRT-PCR in NOK1 and six HNSCC cell lines. Bar: 100 μm. Data are presented as the mean ± S.D. Two-tailed unpaired Student’s t test; *P < 0.05, **P < 0.01

To evaluate the expression profile of m6A “writers”, “erasers”, and “readers” (WERs) in HNSCC, The Cancer Genome Atlas (TCGA) database was analyzed to show that half of the m6A WERs were dysregulated in HNSCC (Fig. 1a). Moreover, AKLBH5 was upregulated and had the highest abundance among the 14 WERs, making it an ideal research object in HNSCC (Fig. 1b). A dot blot assay using an m6A antibody was performed and revealed that the m6A level was lower in HNSCC tissues and cell lines (Fig. 1c, d). The percentage of m6A modification downregulated in HNSCC was compared with that in paired normal tissues using an m6A RNA Methylation Quantification Kit (Fig. 1e). The m6A modification level was lower in most HNSCC cell lines than in primary oral keratinocytes (Fig. 1f). We speculated that the downregulation of m6A in HNSCC may be attributed to demethylase overexpression. On the basis of the expression profile results, we then evaluated the expression of the demethylase ALKBH5 in samples from 138 HNSCC patients and 20 normal oral mucosal samples using immunohistochemistry (Fig. 1g). We found the upregulation of ALKBH5 in HNSCC tissues (Fig. 1h), and its higher expression correlated with advanced TNM stage and poor prognosis in HNSCC but not with other parameters (Fig. 1i, j and Supplementary Fig. S1a-d). ALKBH5 had a sensitivity of 65.7% for HNSCC diagnosis according to the receiver operating characteristic (ROC) curve (Supplementary Fig. S1e). In addition, ALKBH5 was highly expressed in the majority of human cancers, including HNSCC (Supplementary Fig. S1f).

To date, only two m6A demethylases, ALKBH5 and FTO, have been identified. Therefore, we also evaluated the expression of another m6A demethylase, FTO, in samples from 138 HNSCC patients and 20 normal oral mucosal samples using immunohistochemistry. Interestingly, FTO was also upregulated in HNSCC tissues, and its overexpression correlated with advanced TNM stage and poor prognosis in HNSCC (Supplementary Fig. S2a-i). FTO was highly expressed in half of human cancers, including HNSCC (Supplementary Fig. S2j). Consistent with the above results, ALKBH5 protein expression was higher in five tumor tissues than in the adjacent normal oral epithelium tissues (Fig. 1k). Similarly, HNSCC cell lines had higher protein and mRNA expression than normal oral keratinocytes (NOKs) (Fig. 1l, m). These results indicate that upregulated m6A demethylase promotes low m6A status and is correlated with poor prognosis in HNSCC.

### ALKBH5 inhibition decreases cell proliferation *in vitro* and tumor growth *in vivo*

**Fig. 2:**
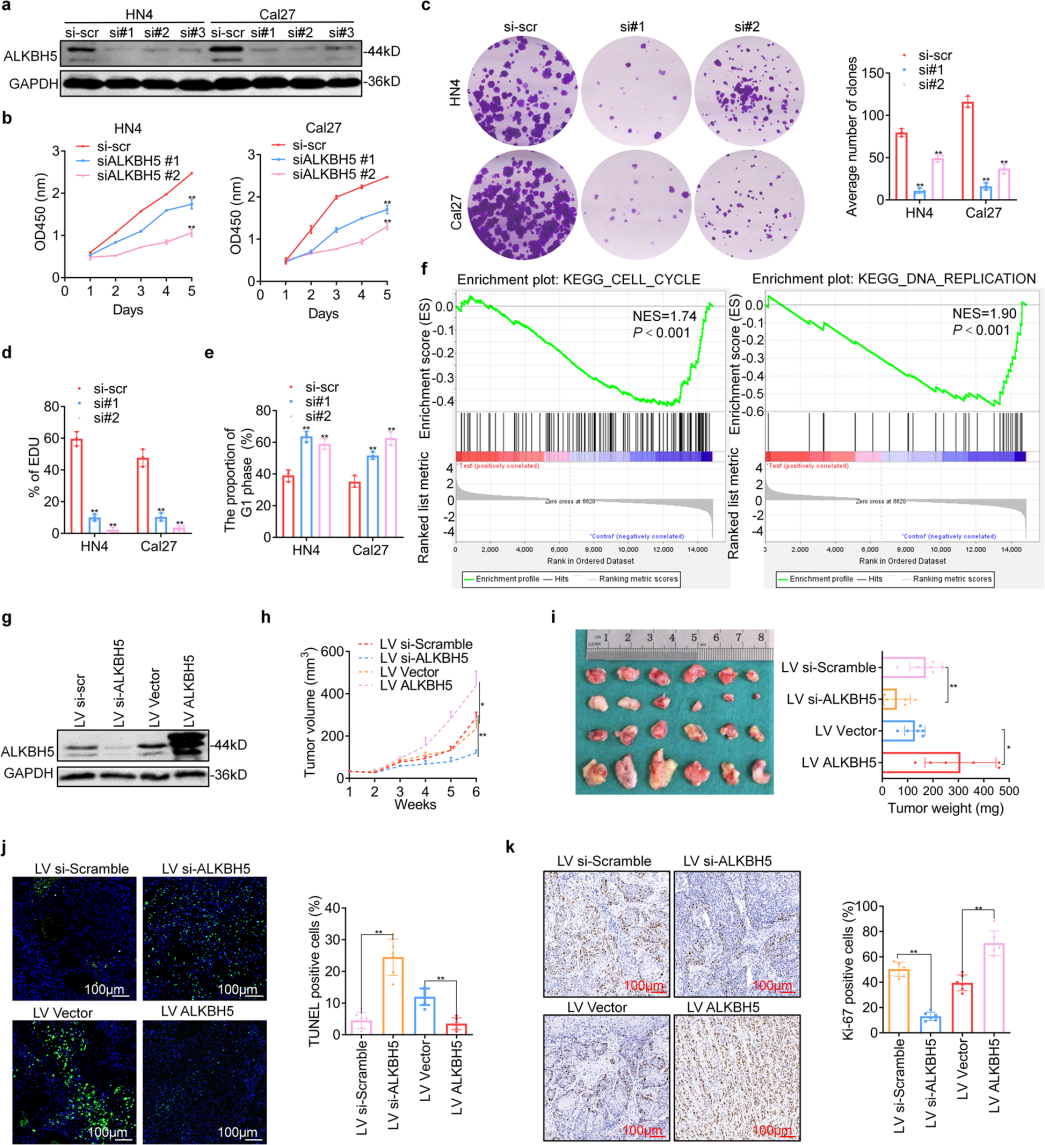
ALKBH5 inhibition decreases cell proliferation *in vitro* and tumor growth *in vivo*. **a** The knockdown efficiency was detected using immunoblotting after siRNA transfection for 48 h. **b**, **c** Cell proliferation and colony formation assays were performed. **d** The EdU assay was performed after siRNA transfection for 48 h. **e** The cell cycle distribution was analyzed after transfection for 48 h using flow cytometry (PI staining). **f** Gene set enrichment analysis (GSEA) plots evaluating the alterations in the cell cycle and DNA replication using RNA sequence data after ALKBH5 knockdown in the Cal27 cell line. NES, normalized enrichment score. **g** The transfection efficiency was detected after lentivirus transduction of ALKBH5 in Cal27 cells. **h** Tumor growth was measured once a week in tumor-bearing nude mice stably transfected with Cal27 cells. **i** The tumors were removed, and the tumor weight was analyzed after the mice were killed. **j**, **k** TUNEL staining and KI-67 staining were performed in tumor tissues from xenografts. Scale bar: 100 μm, Data information: Data are presented as the mean ± S.D. One-way ANOVA; *P < 0.05, **P < 0.01

To explore the role of ALKBH5 in tumor development, we knocked down ALKBH5 expression in HN4 and Cal27 cells using small interfering RNA (siRNA) (Fig. 2a and Supplementary Fig. S3). ALKBH5 inhibition significantly reduced proliferation (Fig. 2b) and colony formation (Fig. 2c) in cell lines. In addition, silencing ALKBH5 reduced DNA replication activity, as shown using an EdU assay (Fig. 2d and Supplementary Fig. S4). The proportions of G1 phase and apoptotic cells also notably increased after ALKBH5 knockdown (Fig. 2e and Supplementary Fig. S5. S6). Kyoto Encyclopedia of Genes and Genomes (KEGG) enrichment analysis of genes with changes in expression after ALKBH5 silencing pointed to cell cycle and DNA replication processes (Fig. 2f). Furthermore, ALKBH5 inhibition suppressed migration and invasion (Supplementary Fig. S7). These results suggested that ALKBH5 acts as an oncogene driving HNSCC development.

To determine the function of ALKBH5 in tumor growth, knockdown and overexpression models were constructed in Cal27 cell lines using lentivirus (Fig. 2g). We showed that knockdown of ALKBH5 expression inhibited tumor growth and weight, while overexpression promoted tumor growth (Fig. 2h, i). Moreover, knockdown of ALKBH5 expression increased cell apoptosis, as revealed by TUNEL staining, while overexpression decreased apoptosis (Fig. 2j). The level of Ki-67, a proliferation marker, decreased after ALKBH5 knockdown and vice versa (Fig. 2k). The above results indicate that ALKBH5 plays a fundamental role in HNSCC development.

### Characterization of m6A modification and analysis of downstream targets of ALKBH5 in HNSCC

**Fig. 3:**
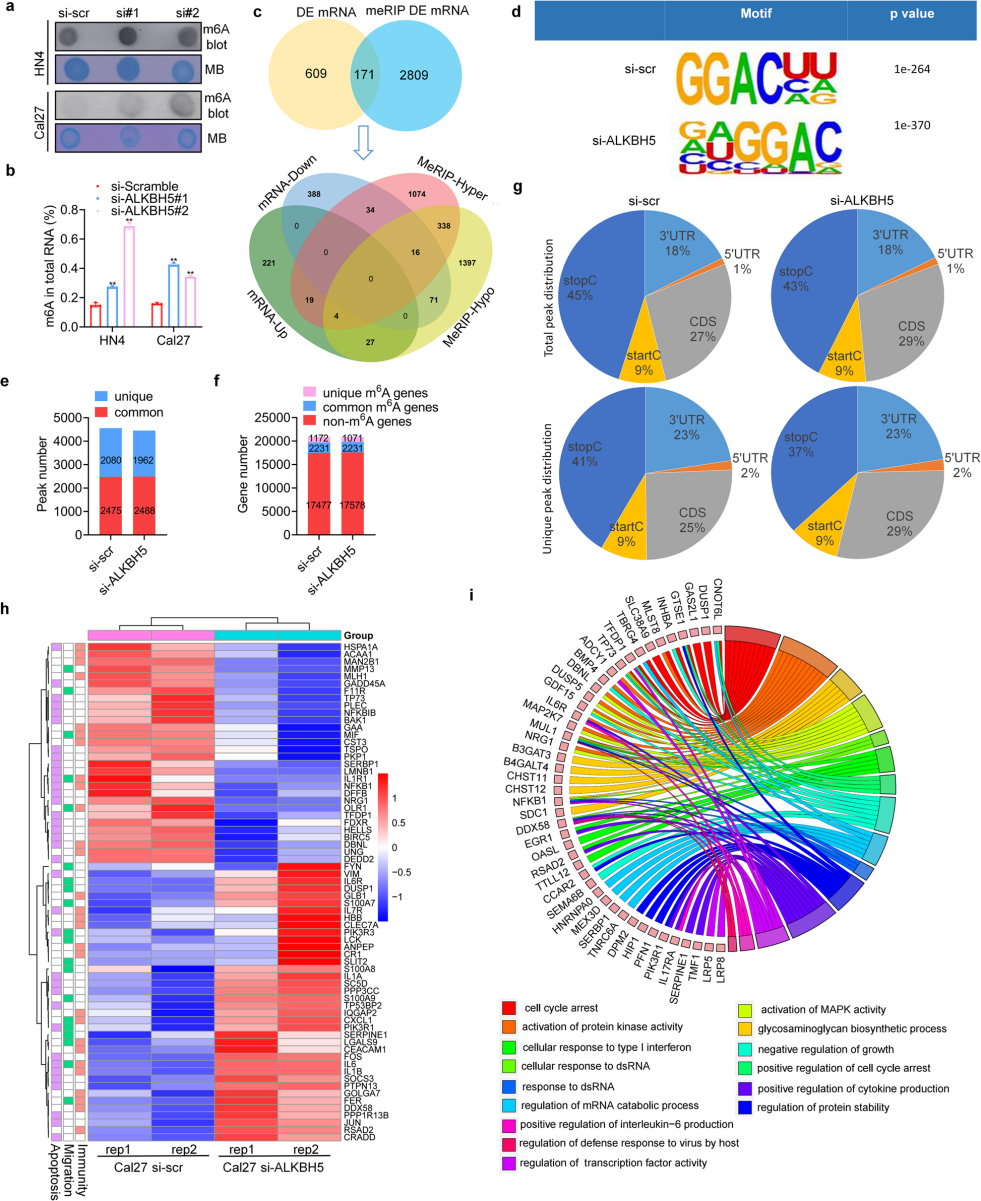
Characterization of m6A modification and analysis of downstream targets of ALKBH5 in HNSCC. **a**, **b** Dot blot and m6A RNA methylation quantification analyses were performed after ALKBH5-specific siRNA transfection for 48 h. **c** Venn diagrams showing 171 genes with differential expression genes and differential m6A-methylation in si-ALKBH5 cells compared with control Cal27 cells (top). A total of 171 common differential genes were classified according to the level of mRNA and m6A methylation (bottom). **d** Predominant consensus motifs identified by HOMER with m6A-seq peaks in Cal27 cells with or without ALKBH5 knockdown. **e**, **f** Number of m6A peaks and m6A-modified genes identified in m6A-seq in si-ALKBH5 and si-scr cells. Unique m6A genes containing no common m6A peaks. **g** Graphs of the m6A peak distribution showing the proportion of total m6A peaks in the indicated regions (top) and the unique m6A peak distribution after ALKBH5 knockdown (bottom). **h** Heatmap showing the expression profile of differentially methylated genes after ALKBH5 knockdown in Cal27 cells with two replicates. **i** The GO terms are visualized in a chord plot. *P < 0.05, **P < 0.01

To understand the mechanism of ALKBH5 in gene expression, both transcriptome and epitranscriptome sequencing were conducted. ALKBH5 depletion increased the total m6A abundance in HNSCC cell lines (Fig. 3a, b). Methylated RNA immunoprecipitation (MeRIP) with an m6A-specific antibody followed by RNA sequencing (MeRIP-seq) and conventional RNA sequencing were combined to analyze the downstream targets of ALKBH5 in Cal27 cells. A total of 171 genes that were differentially expressed and differentially m6A-modified were screened (Fig. 3c and Supplementary Fig. S8). We mapped the m6A motif with independent biological replicates. The GGACU motif was identified to be highly enriched within the m6A site, consistent with the m6A consensus sequence RRACH (R = G or A; H = A, C or U) (Fig. 3d). In general, m6A-seq of control and ALKBH5-deficient cells identified 2080 and 1962 unique m6A peaks, respectively, and 1172 and 1071 unique m6A-modified genes, respectively (Fig. 3e, f). We further investigated the m6A distribution patterns within both the total and unique peaks. Similar patterns of total and common m6A distribution were observed in control and ALKBH5-deficient cells. Interestingly, a relative increase in m6A in coding sequences (CDSs) was observed in an ALKBH5-dependent manner (Fig. 3g).

To explore the biological processes downstream of ALKBH5 silencing, the differentially expressed genes (DEGs) were analyzed by functional enrichment analysis. The DEGs were analyzed and grouped using the cluster analysis method into apoptosis, migration, and immunity (Fig. 3h). Gene Ontology (GO) analysis showed that the DEGs were associated with tumor development processes, such as negative regulation of growth and cell cycle arrest, and innate immunity processes, such as cellular response to type I interferon and response to dsRNA (Fig. 3i).

### ALKBH5 regulates RIG-I expression through m6A modification

**Fig. 4:**
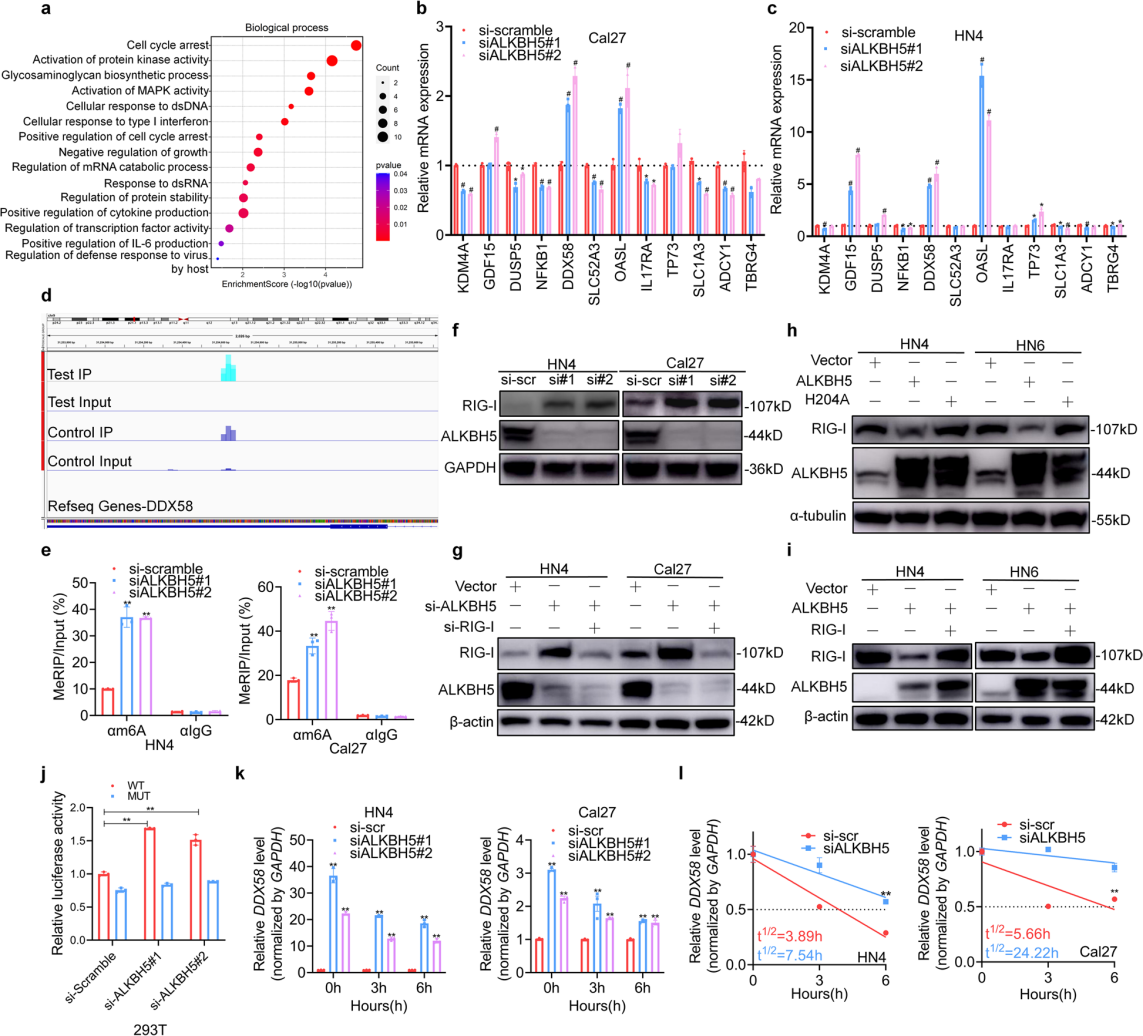
ALKBH5 regulates RIG-I expression through m6A modification. **a** The top 15 biological processes are shown with a bubble chart. **b**, **c** Candidate differentially expressed genes were detected in Cal27 and HN4 cells after ALKBH5 silencing using qRT-PCR. **d** The m6A peak visualization of m6A-seq in DDX58 transcripts in Cal27 cells with or without ALKBH5 depletion is shown. The m6A peaks are in the 3’UTR of DDX58. **e** Methylated RNA in cells with or without ALKBH5 depletion was immunoprecipitated with an m6A antibody, followed by qPCR analyses with primers against DDX58 mRNA. (f) RIG-I, encoded by *DDX58,* was detected by immunoblotting assays after ALKBH5 silencing for 48 h. **g** RIG-I expression was detected by immunoblotting assays after siRNA cotransfection for 48 h. **h** RIG-I expression was detected by immunoblotting assays after ALKBH5 or H204 mutant transfection for 48 h. **i** RIG-I protein expression was detected after plasmid cotransfection for 48 h. **j** Luciferase vectors with the wild-type (WT) or mutated m6A nucleotides (MT) in the *DDX58* gene were transfected into 293 T cells after ALKBH5 depletion. Relative luciferase activity was measured. **k** *DDX58* mRNA expression was detected after incubation with 5 μg/mL actinomycin D for the indicated times and normalized to *GAPDH*. **l** The mRNA half-lives were estimated according to linear regression analysis after the indicated actinomycin D treatment. Data information: Data are presented as the mean ± S.D. One-way ANOVA; *P < 0.05, **P < 0.01

A bubble chart that showed the enrichment of the biological process of interest using the DEGs obtained by sequencing was created (Fig. 4a). Then, the expression of the DEGs of interest in the bubble chart, including those involved in cellular response to type I interferon, were verified using qPCR after ALKBH5 silencing in two cell lines. The increase in DDX58 after ALKBH5 knockdown was the most significant and was relatively consistent between the cell lines (Fig. 4b, c). Volcano plots showed that DDX58 had relatively high mRNA expression and m6A methylation compared to the control (Supplementary Fig. S9). Other genes had similar patterns of expression/methylation as *DDX58*, as presented in the Supplementary Table. S2. Visualization analysis indicated notable m6A enrichment in the 3′-untranslated regions (UTRs) of DDX58 mRNA (Fig. 4d). MeRIP-PCR using an m6A-specific antibody confirmed the enrichment of m6A in the 3’UTR after ALKBH5 knockdown in HN4 and Cal27 cell lines (Fig. 4e). These results indicated that DDX58 was a target of m6A methylation mediated by ALKBH5 in HNSCC.

*DDX58* encodes the protein RIG-I, which is a cytoplasmic receptor that recognizes viral RNA and plays a critical role in innate immunity and type I interferon production. We found that ALKBH5 knockdown significantly increased the protein and mRNA expression of RIG-I (Fig. 4f and Supplementary Fig. S10). Knockdown of RIG-I expression reversed this effect (Fig. 4g). Conversely, the overexpression of wild-type (WT) ALKBH5 but not mutant H204A decreased RIG-I expression (Fig. 4h), indicating that ALKBH5 affected RIG-I expression through its demethylation activity. Similarly, overexpression of RIG-I reversed its ALKBH5-mediated inhibition (Fig. 4i). Moreover, ALKBH5 overexpression inhibited IFNα secretion mediated by RIG-I and vice versa (Supplementary Fig. S11). To determine the effect of ALKBH5-dependent m6A regulation on RIG-I expression, we constructed a luciferase reporter gene with the 3’UTR from DDX58 mRNA, in which the two adenosine (A) bases within the m6A consensus sequences were mutated to cytosine (C) (Supplementary Materials). Luciferase assays showed that ALKBH5 depletion largely increased the activity of luciferase with WT DDX58 but not mutated DDX58 (Fig. 4j). To analyze the effect of the m6A level on DDX58 mRNA metabolism, RNA stability assays were conducted. The assays showed that forced knockdown of ALKBH5 enhanced the expression of DDX58 and prolonged the half-life of DDX58 mRNA transcripts in HNSCC cells (Fig. 4k, l). Thus, the ALKBH5-induced increase in DDX58 is at least in part due to the increased stability of DDX58 mRNA transcripts. These results suggest that ALKBH5-mediated m6A level upregulation of DDX58 mRNA suppresses RIG-I expression.

### RIG-I overexpression reverses the protumorigenic effects of ALKBH5

**Fig. 5:**
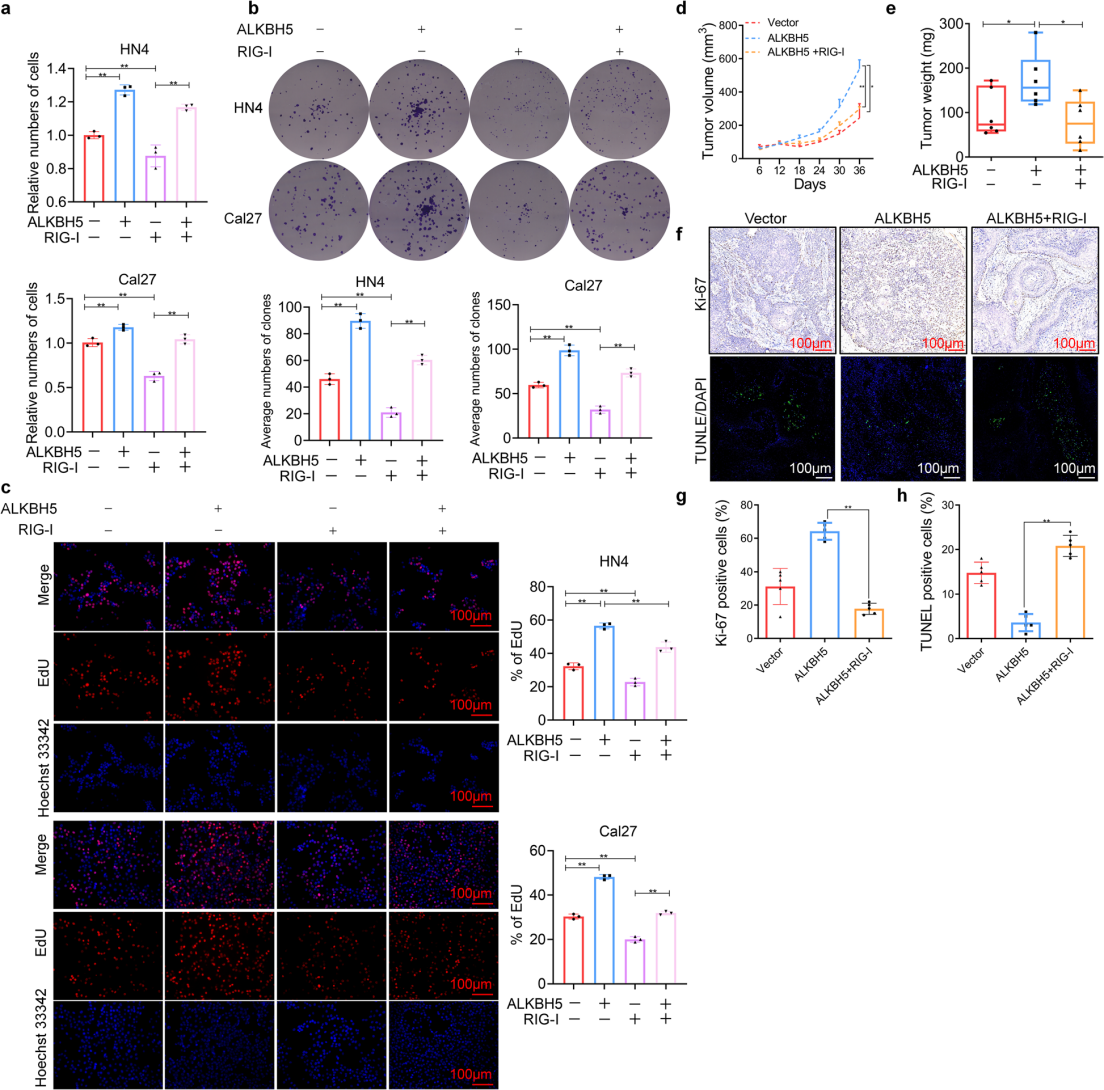
RIG-I overexpression reverses the protumorigenic capacity of ALKBH5. **a** Cell proliferation was detected after transfection with ALKBH5 and/or RIG-I for 3 days. **b** Colony formation assays were performed. **c** EdU assays were performed after transfection with ALKBH5 and/or RIG-I for 48 h. **d**, **e** Tumor volume and weight were measured in nude mouse xenograft models established using lentivirus-transduced cells. **f**, **g**, **h** Ki-67 and TUNEL staining was performed using tumor tissues from the xenograft model. Bar: 100 μm. Data are presented as the mean ± S.D. One-way ANOVA; *P < 0.05, **P < 0.01

As RIG-I is a novel downstream target of ALKBH5, it was essential to explore the role of RIG-I in the protumorigenic effects of ALKBH5. Our previous study fully demonstrated that RIG-I in HNSCC acts as a tumor suppressor *in vitro* and *in vivo* [21]. As expected, the ectopic expression of RIG-I reversed the cell proliferation and increase in cell colony number mediated by ALKBH5 overexpression in HN4 and Cal27 cells (Fig. 5a, b). In addition, an EdU assay showed that RIG-I could decrease the DNA replication activity mediated by ALKBH5 (Fig. 5c). To further support the *in vitro* results, we subcutaneously injected Cal27 cells with or without ALKBH5 or with or without combined ALKBH5 and RIG-I expression into athymic nude mice. We found that overexpression of ALKBH5 increased the tumor size and weight, which were inhibited by RIG-I expression (Fig. 5d, e and Supplementary Fig. S12). Moreover, the increase in the Ki-67 index and the decrease in the TUNEL-positive percentage in the ALKBH5-expressing group were alleviated by combination with RIG-I expression (Fig. 5f, g, h). These results indicate that suppression of RIG-I expression is a key event in ALKBH5-mediated tumorigenesis in HNSCC.

### HNRNPC binds to the ALKBH5-mediated m6A modification of *DDX58* mRNA

**Fig. 6:**
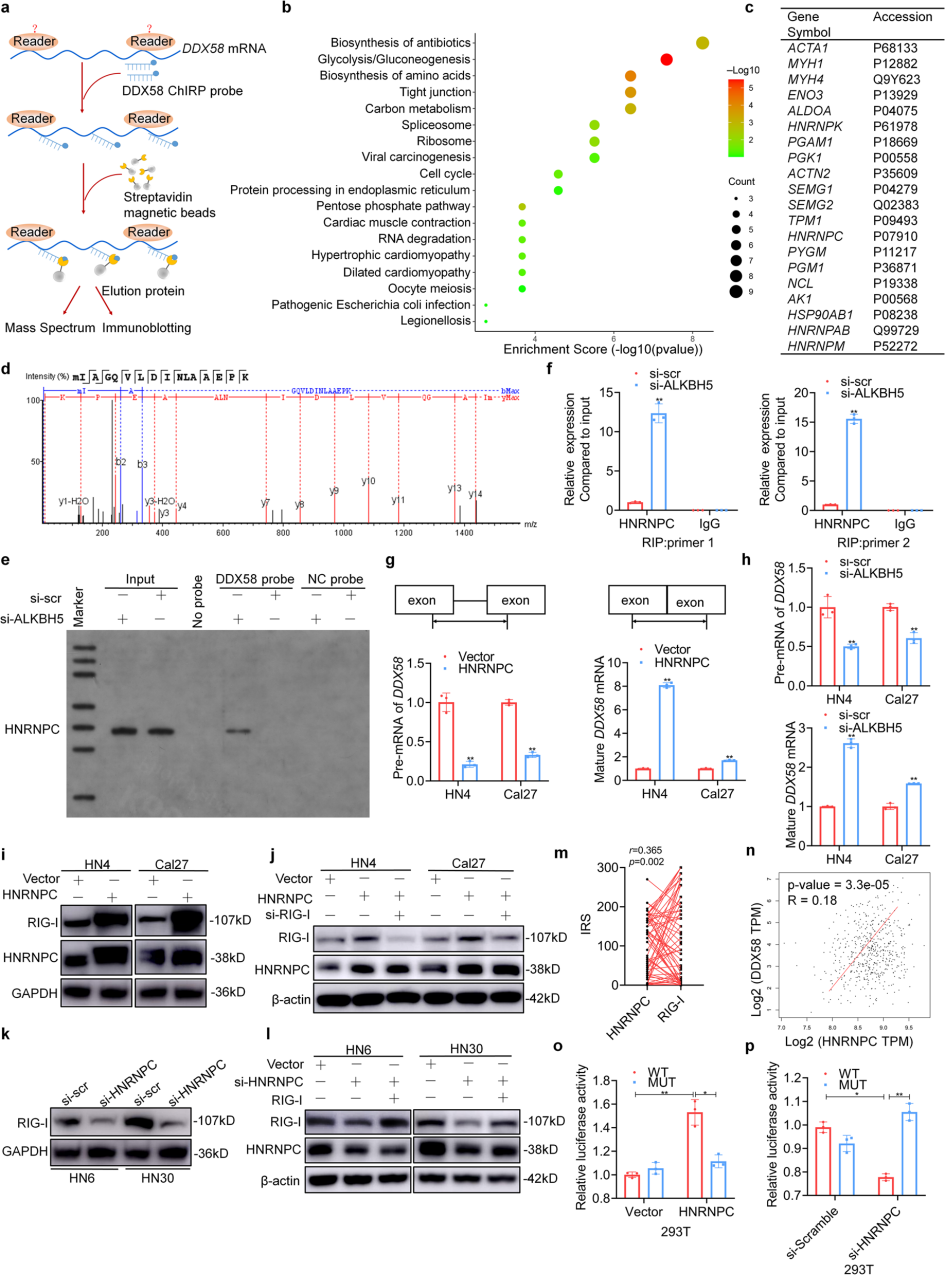
HNRNPC binds to the ALKBH5-mediated m6A modification of DDX58 mRNA. **a** The flowchart of ChIRP assay is shown using biotin-labeled *DDX58* mRNA probes. **b** The bubble chart shows the Kyoto Encyclopedia of Genes and Genomes (KEGG) enrichment analysis of the immunoprecipitated proteins from ChIRP coupled with mass spectrometry. **c** The top 20 precipitated proteins are shown in the table. **d** The identification of HNRNPC by mass spectrometry is shown. **e** The immunoprecipitated proteins from ChIRP were detected using immunoblotting with an anti-HNRNPC antibody. **f** RNA binding protein immunoprecipitation (RIP) assays were performed using an HNRNPC antibody or IgG after si-ALKBH5 transfection for 48 h. Two primers targeting the m6A-modified region of *DDX58* mRNA were used in the RIP-PCR. **g**, **h** Pre-mRNA and mature DDX58 mRNA were analyzed using qPCR after the ectopic expression of HNRNPC or ALKBH5 silencing for 48 h. **i** RIG-I was detected by immunoblotting assays after transfection with HNRNPC expression plasmids for 48 h. **j** RIG-I expression was detected after cotransfection with HNRNPC expression plasmids and siRNA for RIG-I. **k** RIG-I was detected after transfection with siRNA for HNRNPC for 48 h. **l** RIG-I expression was detected after cotransfection with HNRNPC- and RIG-I-siRNA expressing plasmids for 48 h. **m**, **n** The correlations between HNRNPC and DDX58 mRNA or RIG-I protein were analyzed in the HNSCC tissue microarray and TCGA dataset. **o**, **p** Luciferase vectors with the wild-type (WT) or m6A nucleotide-mutated (MT) *DDX58* gene were transfected into 293 T cells with HNRNPC overexpression or depletion. Relative luciferase activity was measured. Data information: Data are presented as the mean ± S.D. Two-tailed unpaired Student’s t test; *P < 0.05, **P < 0.01

It is widely known that the specific role of m6A “readers” in controlling the fate of methylated mRNAs is critical for m6A-dependent biological processes. To elucidate the specific m6A readers of *DDX58* and determine the m6A-dependent mechanism of *DDX58* regulation, ChIRP-MS was conducted using random *DDX58* mRNA-specific biotin-labeled probes (Fig. 6a). Precipitated proteins were analyzed using a bubble chart, and spliceosome, ribosome, and RNA degradation were highlighted as three items involved in m6A-related mRNA metabolism (Fig. 6b and Supplementary Fig. S13). A total of 109 proteins were identified. The top 20 precipitated proteins are shown in Fig. 6c. We identified six unique peptides among seven heterogeneous nuclear ribonucleoprotein C (HNRNPC) peptides, which ranked as having the highest specificity (Fig. 6d). Furthermore, western blot analysis showed that the DDX58 probe bound notably to HNRNPC after ALKBH5 silencing and increased the m6A status (Fig. 6e). Conversely, an RNA binding protein immunoprecipitation (RIP) assay using two specific primers showed that the HNRNPC antibody bound to the 3’UTR of DDX58 mRNA after ALKBH5 inhibition (Fig. 6f). HNRNPC is associated with premRNAs in the nucleus and influences premRNA processing, transport, stability, and other aspects of mRNA metabolism. Therefore, primers designed with or without introns were used to detect premRNA and mature RNA to analyze the processing and maturation function of HNRNPC. We found that overexpression decreased the premRNA level and increased the level of mature *DDX58* mRNA, while ALKBH5 silencing promoted the maturation of *DDX58* mRNA (Fig. 6g, h).

To characterize the role of the binding of HNRNPC to *DDX58* mRNA in RIG-I expression, we evaluated the ectopic expression of HNRNPC and found that it significantly increased the expression of RIG-I (Fig. 6i). RIG-I knockdown reversed this HNRNPC overexpression-mediated effect (Fig. 6j). In contrast, the efficient knockdown of HNRNPC expression using siRNA inhibited RIG-I expression (Fig. 6k and Supplementary Fig. S14). Similarly, RIG-I overexpression reversed the inhibition mediated by HNRNPC silencing (Fig. 6l). Moreover, we observed a positive correlation between HNRNPC and DDX58 or RIG-I in the GEPIA database and in our HNSCC TMA (Fig. 6m, n). In addition, luciferase assays showed that HNRPNC overexpression greatly increased the luciferase activity driven by the WT *DDX58* 3’UTR (containing m6A) but not the mutated *DDX58*, and vice versa (Fig. 6o, p). These results indicated that ALKBH5 silencing increased the m6A modification of *DDX58* mRNA and the subsequent binding of HNRNPC, which promoted the maturation of *DDX58* mRNA and enhanced RIG-I protein expression.

### RIG-I regulated by ALKBH5 affects IFNα secretion through the IKKε/TBK1/IRF3 pathway

**Fig. 7:**
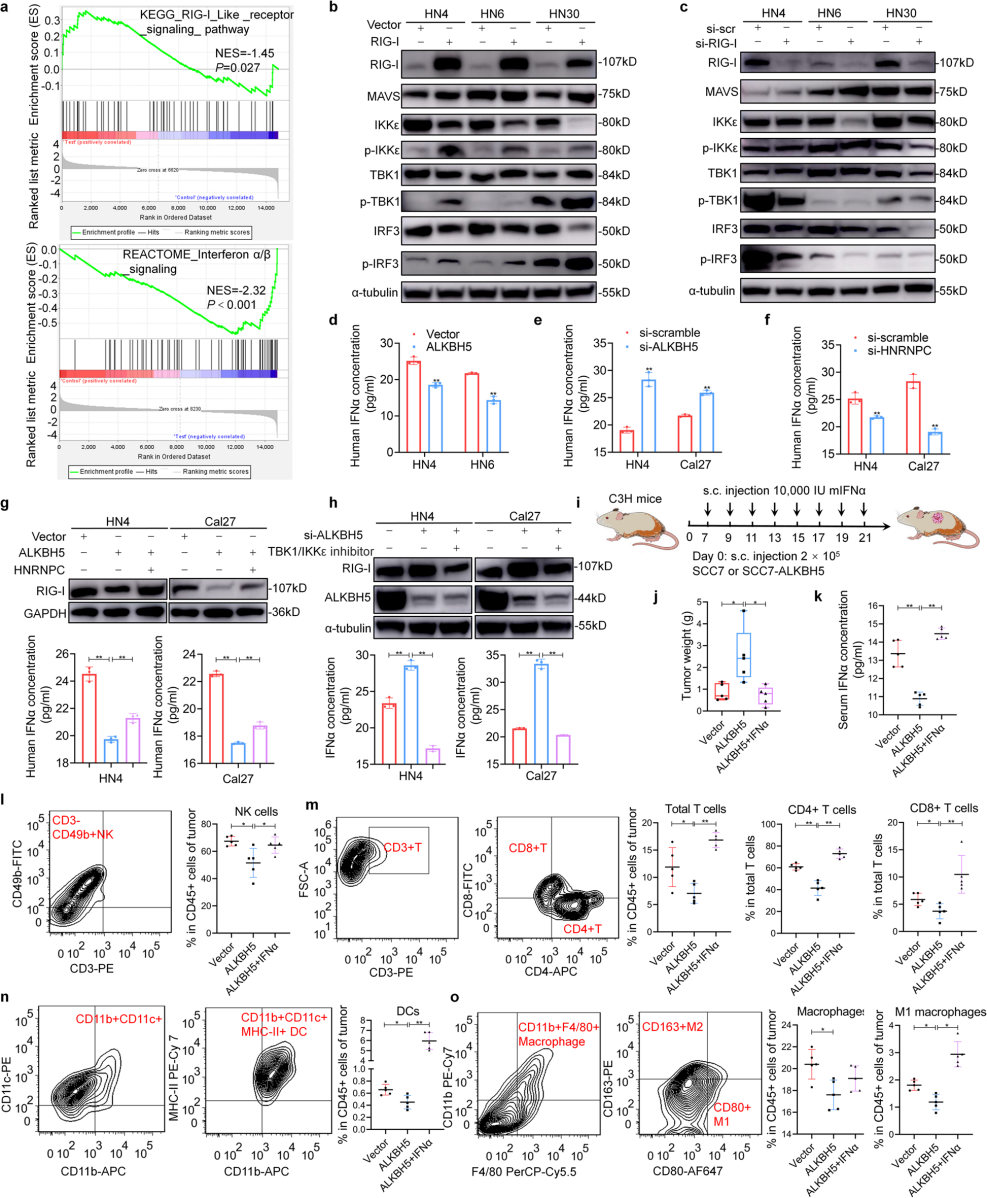
RIG-I regulated by ALKBH5 affects IFNα secretion through the IKKε/TBK1/IRF3 pathway. **a** Gene set enrichment analysis (GSEA) showed the signaling pathways enriched after ALKBH5 silencing. **b**, **c** The activity of the MAVS/IKKε/TBK1/IRF3 signaling pathway was detected after RIG-I overexpression or silencing in head and neck squamous cell carcinoma (HNSCC) cell lines. **d**, **e** The IFNα concentration was measured using an enzyme-linked immunosorbent assay (ELISA) after ALKBH5 overexpression or silencing for 48 h. **f** Supernatant IFNα was detected using ELISA after si-heterogeneous nuclear ribonucleoprotein protein (HNRNPC) transfection for 48 h. **g** The RIG-I protein and IFNα concentrations were analyzed after ALKBH5 and/or HNRNPC transfection for 48 h using immunoblotting and ELISA, respectively. **h** After si-ALKBH5 transfection and TBK1/IKKε-specific inhibitor (10 μM Bay-985) treatment, RIG-I protein and IFNα concentrations were analyzed using immunoblotting and ELISA, respectively. **i** SCC7-bearing xenografts were established in immunocompetent C3H mice, and murine IFNα (mIFNα) was administered at the indicated times and doses. **j** The tumor weight was measured after tumor removal in mice. **k** The serum IFNα concentration was determined using ELISA after mice were sacrificed. **l**-**o** Tumor-infiltrating lymphocytes, including NK, T, DC and macrophage cells, were analyzed by flow cytometry after ALKBH5 silencing and mIFNα injection. Data information: Data are presented as the mean ± S.D. Two-tailed unpaired Student’s t test or one-way ANOVA; *P < 0.05, **P < 0.01

RIG-I recognizes viral RNA, recruits the mitochondrial antiviral signal protein (MAVS), and then activates the IKKε/TBK1/IRF3 pathway to induce type I interferon production. To explore whether ALKBH5 could also affect IFNα secretion through RIG-I regulation, the downstream signaling of RIG-I and IFNα production were detected. Interestingly, gene set enrichment analysis (GSEA) of our RNA sequencing data revealed significant enrichment of the RIG-I-like receptor pathway and interferon α/β signaling after ALKBH5 silencing (Fig. 7a). This enrichment suggests that ALKBH5 is involved in RIG-I signaling and interferon production. Increased phosphorylation of IKKε/TBK1/IRF3 after RIG-I overexpression in HNSCC cell lines was then observed (Fig. 7b). RIG-I silencing inhibited the activation of this pathway (Fig. 7c). Enzyme-linked immunosorbent assays showed increased secretion of IFNα in culture medium after RIG-I overexpression and vice versa (Supplementary Fig. S15). Moreover, we found that ectopic expression of ALKBH5 reduced IFNα secretion, while knockdown of ALKBH5 increased IFNα secretion (Fig. 7d, e). Similarly, silencing of HNRNPC expression also reduced IFNα secretion (Fig. 7f). HNRNPC overexpression reversed the inhibition of RIG-I expression and the subsequent IFNα secretion mediated by ALKBH5 overexpression (Fig. 7g). To explore the role of the IKKε/TBK1/IRF3 pathway, a specific IKKε/TBK1 inhibitor, bay-985, was used. This inhibitor reduced the upregulation of RIG-I expression and IFNα secretion after ALKBH5 knockdown (Fig. 7h).

As a well-known immunomodulatory cytokine, IFNα plays a critical role in the activation of immune cells, such as natural killer (NK) cells, T cells, and dendritic cells (DCs), and exerts an antitumor effect. First, we confirmed the antitumor effect of IFNα in HNSCC cell lines (Supplementary Fig. S16). Then, a xenograft model was established using the murine HNSCC cell line SCC7 in immunocompetent C3H/HeJ mice (Fig. 7i, Supplementary Fig. S17). IFNα reversed the tumor-promoting capacity mediated by ALKBH5 overexpression *in vivo* (Fig. 7j). The serum IFNα concentration was decreased after ALKBH5 overexpression (Fig. 7k). The tumor suppressive function of IFNα was demonstrated in our previous study [21, 24], and it could promote the infiltration of immune-killing cells in the tumor microenvironment (Supplementary Fig. S18). Moreover, the percentages of NK cells, T cells (CD8+ T and CD4+ T), DCs and M1 macrophages among tumor-infiltrating lymphocytes were significantly reduced after ALKBH5 silencing, and this pattern was reversed after murine IFNα injection (Fig. 7l-o and Supplementary Fig. S19). These results indicated that overexpression of ALKBH5 in HNSCC could suppress IFNα secretion through RIG-I regulation and then inhibit immune infiltration and promote tumor progression.

### Upregulated AKLBH5 negatively correlates with RIG-I and IFNα expression in HNSCC patients

**Fig. 8:**
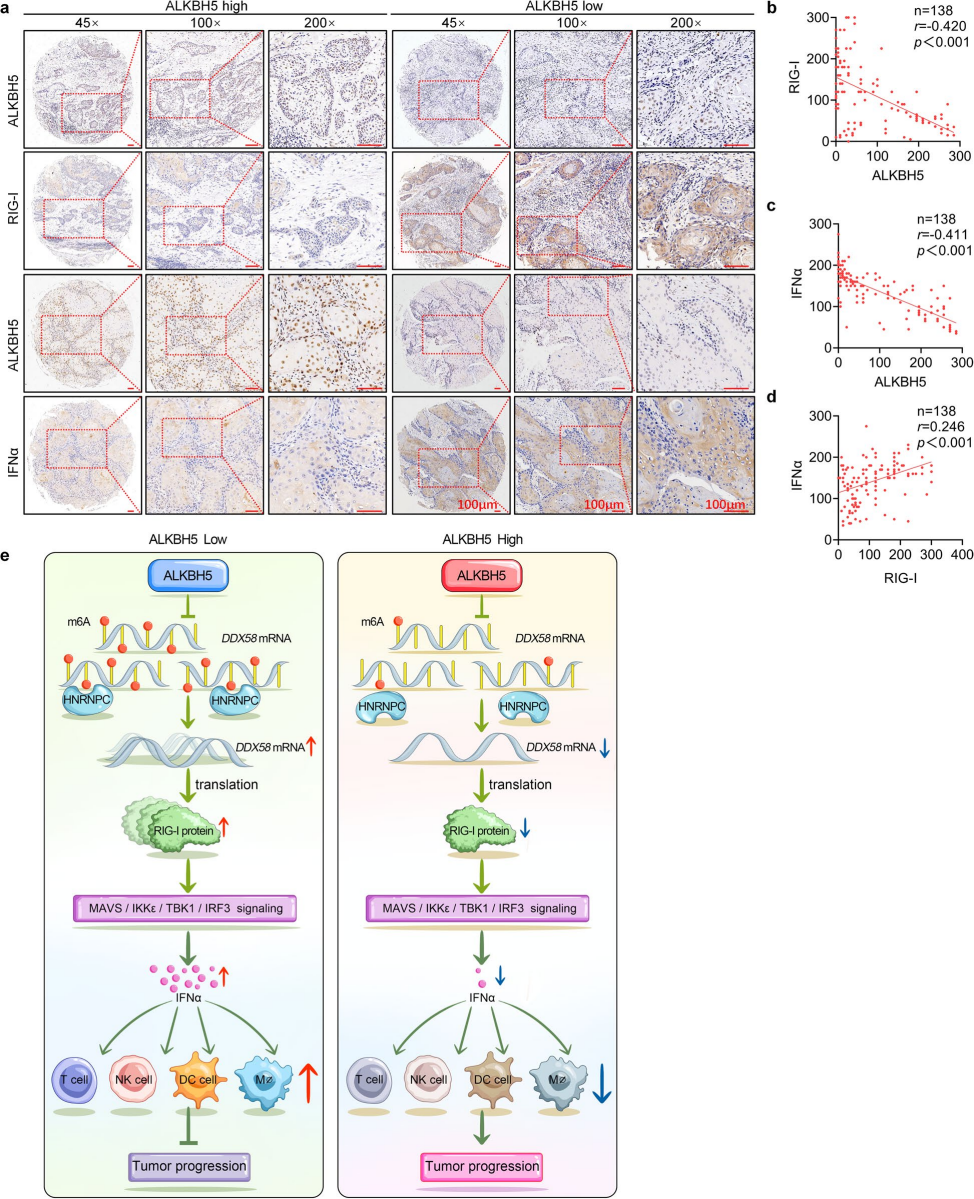
AKLBH5 upregulation is negatively correlated with RIG-I and IFNα expression in HNSCC patients. **a** Representative images of immunohistochemical staining for ALKBH5, RIG-I, and IFNα on a TMA composed of 138 HNSCC tissues. **b**-**d** The correlations among ALKBH5 expression, RIG-I protein expression, and IFNα protein expression were detected and analyzed in a TMA with samples from 138 HNSCC patients. **e** A schematic showing the mechanism by which ALKBH5 upregulation promotes HNSCC progression and immune evasion by inhibiting RIG-I-mediated IFNα secretion via the IKKε/TBK1/IRF3 pathway, ultimately reducing immune-killing cell infiltration in the tumor microenvironment. Bar: 100 μm, Data information: Pearson correlation coefficient; *P < 0.05, **P < 0.01

To investigate the clinical relevance of the ALKBH5/RIG-I/IFNα regulation axis, immunohistochemistry staining was performed in a tissue microarray with 138 HNSCC specimens (Fig. 8a). An inverse correlation between ALKBH5 expression and RIG-I and IFNα protein levels was observed (Fig. 8b, c). Moreover, there was a positive correlation between RIG-I expression and IFNα expression (Fig. 8d). These results verified the correlation in clinical samples. In summary, upregulated ALKBH5 plays an oncogenic role in HNSCC by inhibiting RIG-I-mediated IFNα secretion via the IKKε/TBK1/IRF3 pathway, which ultimately reduces immune-killing cell infiltration and promotes tumor progression (Fig. 8e).

## Discussion

As one of the most prevalent epigenetic modifications in eukaryotic mRNA, m6A methylation is involved in almost all physiological and pathological processes. However, whether it participates in the progression of HNSCC is largely unknown. In the present study, we demonstrate that ALKBH5 plays a critical oncogenic role in HNSCC. *DDX58* mRNA was identified as a target of ALKBH5 through m6A modification and then recognized by HNRNPC. The ablation of RIG-I expression mediated by ALKBH5 upregulation reduces IFNα secretion through the IKKε/TBK1/IRF3 pathway, which ultimately inhibits the infiltration of immune-killing cells and promotes immune escape. This study elucidates a novel regulatory network in immune escape that bridges m6A modification, cancer progression, and innate immunity in HNSCC. Moreover, this study reveals a new mechanism that regulates IFNα production in the tumor microenvironment.

Disorders of m6A modification are closely related to the occurrence and development of many human diseases, especially cancers such as glioblastoma, breast cancer, gastric cancer, and colorectal cancer [25]. ALKBH5 is the only specific m6A demethylase. In contrast, FTO was first identified as an obesity-related gene by genome-wide association analysis. ALKBH5 has been reported to be dysregulated with either tumor-promoting or tumor-inhibiting roles in various cancer types. ALKBH5 is downregulated in hepatocellular carcinoma, and it can inhibit tumor proliferation and invasion capacity, depending on the posttranscriptional inhibition of LYPD1 [26]. Similarly, ALKBH5 also suppresses tumor progression in pancreatic cancer, lung cancer, and esophageal squamous cell carcinoma, with downstream targets such as PER1, YAP, and WIF-1 [27–29]. However, ALKBH5 has been demonstrated to be an oncogene in glioblastoma through the modification of FOXM1 [30], in breast cancer through m6A demethylation of NANOG [15], and in acute myeloid leukemia via AXL m6A modification [31]. This divergence might be attributable to differences in cell type, tumor heterogeneity, or other unknown factors, which suggests the complexity of m6A in carcinogenesis. From the TCGA HNSCC dataset, it can be seen that the two m6A demethylases are expressed at significantly higher levels in tumor samples than in normal control samples, while ALKBH5 has relatively high abundance in HNSCC [32]. Meanwhile, univariate and multivariate analyses have demonstrated that upregulation of ALKBH5 and YTHDC2 are the only two independent risk factors for overall survival in HNSCC [33]. In our study, high ALKBH5 was correlated with poor prognosis in HNSCC patients, and the correlation was more obvious than that in FTO. It was therefore very reasonable to consider ALKBH5 as the research object in this study. These bioinformatics data support our findings that ALKBH5 plays an oncogenic role in HNSCC and promotes tumor proliferation and migration dependent on m6A modification.

The screening and identification of downstream targets serve as the core of m6A modification research. m6A regulatory proteins play diverse roles in different biological processes, depending on their specific downstream targets. Currently, the identified m6A downstream targets in tumors include many well-known molecules involved in classical signaling pathways, such as FoxM1 [30], β-catenin [34], Sox2 [35], and Notch1 [36]. In contrast to a previous study, our study identified RIG-I, an important natural immune receptor, as an m6A-modified downstream target. The previous study showed that m6A modification is involved in antitumor immunity [37], and RIG-I acts as an m6A modification target participating in the process of antiviral innate immunity [38]. Our study also confirmed that RIG-I is an m6A target in tumor immunity. ALKBH5 regulates downstream signal transduction through the methylation of RIG-I, which ultimately affects the secretion of IFNα in tumor cells. Combined with our previous research, these findings show that IFNα is an important immune-activating factor that plays a key role in DC maturation and migration, T-cell survival, NK-cell cytotoxicity, and macrophage polarization [39]. IFNα is a powerful and effective antitumor immune effector molecule. Our study found that overexpression of ALKBH5 inhibits IFNα secretion through RIG-I, reduces the infiltration of immune-killing cells, and then promotes tumor immune escape. This study answered the question of what regulates IFNα secretion in the tumor microenvironment. Moreover, these findings also connect m6A modification with innate immunity and immune escape, providing a new perspective on the regulation of m6A modification in tumors.

There are several limitations in this study. Specifically, this study mainly focuses on m6A demethylases because to date, only ALKBH5 and FTO have been identified as m6A “erasers”. Although m6A “writers” and “readers” have more family members, this study did not evaluate the expression of all m6A regulators in HNSCC. The selection of ALKBH5 in our study was based on the TCGA HNSCC dataset [32, 33]. ALKBH5 significantly inhibited the expression of RIG-I in this study. However, whether m6A “writers” can regulate its expression in an opposite manner is still unknown.

## Conclusions

In summary, we discovered a novel ALKBH5/RIG-I/IFNα axis that promotes tumor progression by escaping immune killing mediated by the m6A-dependent HNRNPC binding of *DDX58* mRNA in HNSCC. This study not only expands our understanding of m6A-regulated tumor progression from a new perspective, IFNα-mediated innate immunity, but also explains the regulatory mechanism of IFNα secretion in the tumor microenvironment at the posttranscriptional modification level. These findings provide new insights into carcinogenesis and promising approaches for the treatment of HNSCC patients.

## Methods

### Tissue microarray and cell culture

A total of 138 pairs of tissue samples were obtained from patients with HNSCC who underwent radical resection at the Department of Oral Maxillofacial-Head and Neck Oncology, Shanghai Ninth People’s Hospital, Shanghai Jiao Tong University School of Medicine. All tumor and adjacent normal tissues used in this study were collected with informed consent. This study was approved by the Ethics Committee of the Ninth People’s Hospital, Shanghai Jiao Tong University School of Medicine. The HNSCC cell lines HN4, HN6, and HN30 were provided by the University of Maryland. Cal27, SCC4, SCC25, and 293 T cells were purchased from the Type Culture Collection of the Chinese Academy of Sciences (Shanghai, China). SCC7, a mouse-derived HNSCC cell line, was donated by Prof. Liu of Soochow University. Primary NOKs were primarily cultured.

### Dot blot and m6A quantification

Preheated RNA was spotted on a positively charged nylon membrane (RPN303B, Amersham, USA). The m6A status was probed with anti-m6A antibody (ABE572, Millipore, USA) and detected using ECL detection reagents (P36599, Millipore, MA, USA). The same membrane was also stained with methylene blue (319,112, Sigma, St. Louis, MO) as a control. The global m6A levels in mRNA were quantified with an EpiQuik m6A RNA Methylation Quantification Kit (Colorimetric) (Abcam) following the manufacturer’s protocol.

### Immunohistochemistry

After deparaffinization, rehydration and antigen retrieval, slides were incubated with primary rabbit anti-human ALKBH5 (dilution 1:500; Abcam Antibody; ab195377), primary rabbit anti-human RIG-I (dilution 3.75 μg/ml; Abcam Antibody; ab238254) or rabbit anti-human IFNα (dilution 1:100; Thermo Fisher; PA5–115430) at 4 °C. We quantitatively scored the tissue slides according to the percentage of positive cells and staining intensity. The H-score (maximum score, 300) was assessed using the following formula: (3 × percentage of strongly staining) + (2 × percentage of moderately staining) + percentage of weakly staining.

### Immunoblotting

Immunoblotting was performed as described in our previous study. The antibodies used in this study were as follows: ALKBH5, FTO, RIG-I and HNRNPC purchased from Abcam (Cambridge, MA, UK); MAVS, IKKε, p-IKKε, TBK1, p-TBK1, IRF3, and p-TBK1 purchased from Cell Signaling Technology (Danvers, MA, USA); GAPDH and α-tubulin purchased from Proteintech Inc. (Proteintech, Rocky Hill, NJ, USA). The TBK1/IRF3-specific inhibitor Bay-985 was purchased from Selleck (Houston, TX, USA).

### RNA isolation and quantitative real-time PCR

Total RNA was isolated with TRIzol reagent (Invitrogen, USA), and qRT–PCR was conducted using a StepOnePlus Real-time PCR system (Applied Biosystems, Waltham, MA, USA) following the manufacturer’s instructions. The primer sequences are listed in Supplementary Table S1.

### Cell transfection and lentivirus transduction for stable cell lines

Cells were transfected with siRNA using RNAiMAX and plasmids with Lipofectamine 3000 (Invitrogen, USA) according to the manufacturer’s instructions. Lentiviruses expressing ALKBH5 or RIG-I were constructed by Genomeditech Company (Shanghai, China).

### Cell Counting Kit-8, colony formation assay, Transwell assay, 5-ethynyl-2′-deoxyuridine (EdU) assay and terminal deoxynucleotidyl transferase dUTP nick end labeling (TUNEL) assay

Cell proliferation was detected using a Cell Counting Kit (CCK8; Dojindo, Kumamoto, Japan). Cells were seeded into 6-well plates at 500 cells/well for 10 ~ 14 days to analyze the clone-forming capacity. Transwell assays were performed using uncoated polycarbonate inserts (Millipore, Darmstadt, Germany) to test migration or BioCoat™ inserts (BD Biosciences, Franklin Lake, NJ, USA) to test invasion. EdU (RiboBio, Guangzhou, China) and TUNEL (Beyotime, Shanghai, China) staining were conducted according to the manufacturers’ protocols.

### Flow cytometry

For apoptosis analysis, Annexin V-FITC/propidium iodide (PI) apoptosis kits (BD Biosciences) were used, and measurements were performed with a BD Beckman cytometer (BD Biosciences, Franklin Lakes, NJ, USA) and FlowJo software. For cell cycle analysis, cells were incubated with PI/RNase staining kit reagents (BD Biosciences, Franklin Lakes, NJ, USA). Anti-mouse CD3e APC and anti-mouse CD49b FITC antibodies were used for NK cell analysis. Anti-mouse CD11b FITC, anti-mouse CD11c PE-Cy7, and anti-mouse I-A/I-E APC antibodies were purchased for DC analysis. Anti-mouse CD8a PE, anti-mouse CD4 FITC, and CD3e APC antibodies were used for T cell analysis. Anti-mouse CD11b PE-Cy7, F4/80 PerCP-Cy5.5, CD80 AF647, and CD163-PE antibodies were used to analyze macrophages. All of the antibodies for immune cells were purchased from Peprotech (Rocky Hill, NJ, USA) and analyzed using a BD Fortessa cytometer (BD Biosciences, Franklin Lakes, NJ, USA).

### Dual-luciferase reporter assays

The DNA fragments of the DDX58 3’UTR containing two m6A motifs, as well as the mutated motifs (m6A was replaced by C), were inserted into a PGL3-CMV-LUC-MCS plasmid vector (Genomeditech, Shanghai, China). The mutation sites are shown in the supplementary materials. Dual-luciferase reporter assays were performed using HEK 293 T cells (Beyotime, Shanghai, China).

### Animal experiments

For the subcutaneous implantation model, 1 × 10^6^ Cal27 cells stably transduced with lentivirus were injected into the left or right flanks of BALB/c nude mice (aged 4–6 weeks). Following stable transfection, 2 × 10^5^ SCC7 cells were subcutaneously inoculated into C3H mice (aged 6–8 weeks), which were purchased from the Shanghai Laboratory Animal Center (Shanghai, China). The width (W) and length (L) of the tumors were measured every week, and the volume was calculated using the formula V = (L × W^2^/2). All animal experiments were approved by the Animal Care and Use Committee of the Ninth People’s Hospital, Shanghai Jiao Tong University School of Medicine.

### Tumor infiltration lymphocyte (TIL) analysis

Established tumors were removed from mice, dissociated, and treated with 0.4 mg/mL collagenase IV, 0.4 mg/ml hyaluronidase, and 30 U/ml DNase I in RPMI 1640 medium containing 10% FBS for 1 h at 37 °C. All reagents were purchased from Sigma. Cells were washed twice, followed by filtration through a 70-μm strainer. The obtained single-cell suspensions were stained with anti-mouse CD45 PerCP-Cyanine 5.5 and Viability Dye 450 to distinguish immune cells and live/dead cells (Peprotech, Rocky Hill, NJ, USA).

### m6A-RIP and m6A sequencing (MeRIP-Seq) assay, RNA sequencing, and sequencing data analysis

MeRIP-Seq and RNA-Seq were performed by Cloudseq Biotech Inc. (Shanghai, China) according to standard procedures. Fragmented mRNA was incubated with an anti-m6A polyclonal antibody (Synaptic Systems, 202,003) in IPP buffer (10 mM Tris HCl, 150 mM NaCl, 0.1% NP40, pH 7.4), which was then immunoprecipitated by incubation with protein-A beads. The bound RNA was eluted from the beads with m6A (Berry & Associates, PR3732) and then extracted with TRIzol reagent (Thermo Fisher, Invitrogen, USA) following the manufacturer’s instructions. Purified RNA was used for RNA sequencing library generation with the NEBNext® Ultra™ RNA Library Prep kit (NEB). The input sample and the m6A IP samples were analyzed with 150 bp paired-end sequencing on an Illumina HiSeq 4000 sequencer.

First, adaptors and low-quality reads were filtered by cutadapt; then, the clean reads were aligned to the reference genome (UCSC HG19) by Hisat2 software (v2.0.4). The m6A peaks were analyzed by Model-based Analysis of ChIP-Seq (MACS) software. For the identification of consensus sequences, motifs were analyzed by HOMER. The metagenes of the m6A regions were generated with the R package MetaPlotR. Differentially methylated sites with a fold-change cutoff of ≥2 and a false discovery rate cutoff of ≤0.00001 were screened by diffReps61. The paired-end, adaptor-cleaned reads mapped to each gene were calculated by HTSeq software (v0.9.1) and normalized by edgeR software. GO and KEGG enrichment analyses were performed on differentially methylated and expressed genes.

### MeRIP-PCR

To detect the m6A modification of target genes, the Magna MeRIP™ m6A Kit (17–10,499, Millipore, Billerica, MA) was used according to the manufacturer’s instructions. Briefly, 300 μg of total RNA was enriched with a monoclonal antibody against m6A.

The RNA of interest was immunoprecipitated with Protein A/G Magnetic Beads in 500 mL of 1x IP buffer supplemented with RNase inhibitors at 4 °C overnight. After immunoprecipitation, isolated RNA fragments were subjected to qRT–PCR. Thirty micrograms of the fragmented RNA sample was saved as the 10% input control and further analyzed.

### ChIRP-MS

Thirty-six biotin-labeled probes targeting DDX58 and a negative probe were directly synthesized by gene synthesis (RiboBio, Guangzhou, China). In brief, 3 × 10^7^ cells were fixed by adding 37% formaldehyde solution at a ratio of 1:36 at room temperature, followed by ultrasonic lysis. Five microliters of biotin-labeled probe was incubated in RNA protein hybridization buffer overnight at room temperature. Fifty microliters of streptavidin magnetic beads were added to immunoprecipitate the RNA-binding proteins. Eluted proteins were separated by SDS–PAGE for electrophoresis, and the bands of interest were excised for mass spectrometry analysis. The selected differentially expressed proteins were annotated with GO and KEGG analyses.

### RIP

RIP analysis was performed using an RNA-binding protein immunoprecipitation kit according to the manufacturer’s instructions (Cat: 17–700, Millipore, Darmstadt, Germany). At 24 h after transfection, the cells were incubated with RIP buffer containing magnetic beads conjugated with IgG and HNRNPC antibodies overnight at 4 °C. The samples were then incubated with proteinase K to isolate immunoprecipitated RNA. The isolated RNA was analyzed by RT–PCR with primers targeting DDX58 truncations (Additional file 1: Table S1).

### Enzyme-linked immunosorbent assay (ELISA)

After transfection or treatment, cell supernatants were collected for detection with a human IFNα ELISA kit (Shycbio, Shanghai, China). Blood was collected from the hearts of mice, and the serum IFNα concentration was detected according to the manufacturer’s instructions (Shycbio, Shanghai, China).

### Statistical analysis

We used unpaired Student’s t tests to compare means between groups and ANOVA for comparisons among more than three groups. All data are expressed as the mean ± SD. The survival analyses were performed using the Kaplan–Meier method to plot survival curves and the log-rank test to compare survival rates. The correlation between ALKBH5 and RIG-I and IFNα levels was analyzed by the Pearson correlation coefficient. *p* values <0.05 were considered to be significant. All statistical tests were two-sided.

## Supplementary Information

**Additional file 1: Supplementary Table S1.** The primers and sequences used in this study.

**Additional file 2: Supplementary Table S2.** The differentially expressed genes with upregulation of mRNA/methylation after sequencing.

**Additional file 3: Supplementary Figure S1.** The correlation between ALKBH5 expression and clinicopathological features in 138 HNSCC patients. (a-d) The correlation between the IRS of ALKBH5 and age, sex, pathological grade and lymph node status was analyzed in 138 HNSCC patients. (e) ROC curves were generated according to the IRS of ALKBH5 in HNSCC and normal controls. (f) The pancancer expression profile of ALKBH5 according to the GEPIA dataset. **Figure S2.** The correlation between FTO expression and clinicopathological characteristics in 138 HNSCC patients. (a) Representative images of immunohistochemical staining for FTO protein on a tissue microarray composed of 138 HNSCC tissues and 20 normal epithelial tissues. Scale bars: 100 μm. (b) The expression level of FTO was analyzed in HNSCC and the normal control. (c) ROC curves were generated according to the IRS of FTO in HNSCC and normal controls. (d-h) The correlation between the IRS of ALKBH5 and age, sex, pathological grade, lymph node status and TNM stage was analyzed in 138 HNSCC patients. (i) The Kaplan–Meier method with a two-tailed log-rank test was used to plot survival curves in The Cancer Genome Atlas (TCGA) HNSCC dataset with high and low FTO expression. The log-rank test was used to compare the survival rate. (j) The expression profile of FTO in pancancer tissues according to the GEPIA dataset. *P < 0.05, **P < 0.01. **Figure S3.** *ALKBH5* mRNA was detected after siRNA transfection for 24 hours. **Figure S4.** The EdU assay was performed after siRNA transfection for 48 hours. **Figure S5.** The cell cycle distribution was analyzed using flow cytometry (PI staining) after transfection for 48 hours. **Figure S6.** Apoptotic cells were detected using flow cytometry (Annexin V/PI staining) after transfection for 48 hours. **Figure S7.** The migration and invasion capacities were detected using Transwell inserts with and without Matrigel, respectively, after transfection for 48 hours. **Figure S8.** Venn diagrams show differentially expressed genes with >2-fold alterations after ALKBH5 knockdown. **Figure S9.** Volcano plots showing the m6A enrichment and mRNA expression levels of genes in ALKBH5-deficient cells compared to the control. **Figure S10.** Relative *DDX58* levels were detected by qRT-PCR after ALKBH5 silencing for 24 h. **Figure S11.** The IFNα concentration in the supernatant was detected after knockdown or overexpression of both ALKBH5 and RIG-I for 48 h. **Figure S12.** The tumors in a xenograft model after ectopic expression of ALKBH5 with or without RIG-I expression are shown. **Figure S13.** Bubble chart showing the KEGG enrichment analysis of the immunoprecipitated proteins from ChIRP coupled with mass spectrometry. **Figure S14.** HNRNPC knockdown efficiency of three siRNAs, detected using immunoblotting. **Figure S15.** IFNα concentration measured using an enzyme-linked immunosorbent assay (ELISA) after RIG-I overexpression or silencing for 48 h. **Figure S16.** The IC50 of IFNα determined after 72 hours in HNSCC cells. **Figure S17.** The representative image of ALKBH5 overexpression and mIFNα treatment in SCC7-bearing C3H mice. **Figure S18.** The infiltration of immune cells in tumor microenvironment was analyzed after mIFNα or PBS treatment in SCC7-bearing xenograft. **Figure S19.** The percentage of M2 macrophage was analyzed in SCC7-bearing C3H mice by flow cytometry.

## Data Availability

All data supporting this study are available within this article and supplementary Information file. The MeRIP-seq and mRNA-seq data have been deposited into the Gene Expression Omnibus repository under accession number GSE185888. You may view the GSE185888 study at: https://www.ncbi. nlm.nih.gov/geo/query/acc.cgi?acc=GSE185888. The dataset used and/or analyzed during the current study are available from the corresponding author on reasonable request.

## Abbreviations

ALKBH5: AlkB homolog 5, RNA demethylase
HNSCC: Head and neck squamous cell carcinoma
m6A: N6-methyladenine
IFNα: Interferon alpha
RIP: RNA-binding protein immunoprecipitation assay
TCGA: The Cancer Genome Atlas
MeRIP: m6A-RNA immunoprecipitation
ChIRP-MS: Chromatin Isolation by RNA Purification coupled Mass Spectrum
METTL3: Methyltransferase-like 3
WTAP: Wilms tumor-associated protein
FTO: Fat-mass and obesity-associated protein
IGF2BPs: Insulin growth factor-2 mRNA-binding proteins
HNRNP: Heterogeneous nuclear ribonucleoprotein
EdU: 5-Ethyny-2′-deoxyuridine
TUNEL: Terminal deoxynucleotidyl transferase dUTP nick end labelling assay
KEGG: Kyoto Encyclopedia of Genes and Genomes
