# Supplementary material for: The m6A demethylase ALKBH5 promotes tumor progression by inhibiting RIG-I expression and interferon alpha production through the IKKε/TBK1/IRF3 pathway in head and neck squamous cell carcinoma
Source: Mol Cancer. 2022 Apr 9;21:97. doi: 10.1186/s12943-022-01572-2 (PMC8994291; doi:10.1186/s12943-022-01572-2)

Supplementary Figures

Fig. S1. The correlation between ALKBH5 expression and clinicopathological features in 138 HNSCC patients. (a-d) The correlation between IRS of ALKBH5 and age, gender, pathological grade and lymph node status was analyzed in 138 HNSCC patients. (e) ROC curve was operated according to the IRS of ALKBH5 in HNSCC and normal controls. (f) The expression profile of ALKBH5 in pan-cancer tissues according to GEPIA dataset.

Fig. S2 The correlation between FTO expression and clinicopathological characteristics in 138 HNSCC patients. (a) Representative images of immunohistochemistry staining for FTO protein on a tissue microarray composed of 138 HNSCC tissues and 20 normal epithelium tissues. Scale bars: 100 μm. (b) The expression level of FTO was analyzed in HNSCC and the normal control. (c) ROC curve was operated according to the IRS of FTO in HNSCC and normal controls. (d-h) The correlation between IRS of ALKBH5 and age, gender, pathological grade, lymph node status and TNM stage was analyzed in 138 HNSCC patients. (i) The Kaplan–Meier method with two-tailed log-rank test was used to plot survival curves in The Cancer Genome Atlas (TCGA) HNSCC dataset with high and low FTO expression. The log-rank test was used to compare the survival rate. (j) The expression profile of FTO in pan-cancer tissues according to GEPIA dataset. **P* < 0.05, ***P* < 0.01.

Fig. S3 The *ALKBH5* mRNA was detected after siRNA transfection for 24 hours.

Fig. S4 The EdU assay was performed after siRNA transfection for 48 hours.

Fig. S5 The cell cycle distribution was analyzed after transfection for 48 hours using Flow cytometry (PI staining).

Fig. S6 The apoptotic cells were detected using flow cytometry (Annexin V / PI staining) after transfection for 48 hours.

Fig. S7 The migration and invasion capacity were detected using Transwell insert with or without Matrigel after transfection for 48 hours.

Fig. S8 Venn diagrams show differential expression genes with ＞2 fold of alterations after ALKBH5 knockdown.

Fig. S9 Volcano plots showing the m6A enrichment and mRNA expression levels of genes in ALKBH5-deficient cells compared to the control.

Fig. S10 Relative DDX58 level was detected by qRT-PCR after ALKBH5 silencing for 24 h.

Fig. S11 The IFNα concentration in supernatant was detected after knockdown or overexpression of both ALKBH5 and RIG-I for 48 h.

Fig. S12 The tumors were shown in xenograft model after ectopic expression of ALKBH5 with or without RIG-I expression.

Fig. S13 The bubble chart showed the enrichment analysis of KEGG using the immunoprecipitated proteins from ChIRP coupled mass spectrum.

Fig. S14 The knockdown efficiency was detected using immunoblotting after three siRNAs targeting HNRNPC.

Fig. S15 The IFNα concentration was measured using an enzyme-linked immunosorbent assay (ELISA) after RIG-I overexpression or silencing for 48 h.

Fig. S16 The IC50 of IFNα was determined after 72 hours in HNSCC cells.

Fig. S17 The representative image of ALKBH5 overexpression and mIFNα treatment in SCC7-bearing C3H mice.

Fig. S18 The infiltration of immune cells in tumor microenvironment was analyzed after mIFNα or PBS treatment in SCC7-bearing xenograft.

Fig. S19 The percentage of M2 macrophage was analyzed in SCC7-bearing C3H mice by flow cytometry.

Supplementary materials

The luciferase reporter plasmids construction contained the WT m6A motif, as well as the mutated motifs (m6A was replaced by C).

DDX58-3’UTR with WT m6A sites:

GTATGTATATTAGTTACATATACATATGTACAGTATATACATGTATATATATTGAACCCTTGGAAGGGATCAGCAAAGAGGA**GGACT**CAATAAAAAA**AGACT**AGAATAGAACTAGAAGAGAGAGAAGCCCAGGGAGAGCTTCAAGAAGAGTGCTCAACAGGGCCAAATGCGCAGAGGTCAAATAGAAGGTTAAGAAGTGCCCAGTGGATTTGGCAACCAGAGTCACTGGTAACCTTCAAAAGAACAATTTCAGTAGATTGTTAAGGGCAAAAGCCAGTTTACAGTAGGTTAAGGAGACAAAGGGAGATAAAAATTCCCAACATTCCATTCATTCATCTATCCCAGTGTGTACAGAGTGATTACTGTGTGTTTGTTGGCAG

DDX58-3’UTR with the mutated m6A sites:

GTATGTATATTAGTTACATATACATATGTACAGTATATACATGTATATATATTGAACCCTTGGAAGGGATCAGCAAAGAGGA**GGCCT**CAATAAAAAA**AGCCT**AGAATAGAACTAGAAGAGAGAGAAGCCCAGGGAGAGCTTCAAGAAGAGTGCTCAACAGGGCCAAATGCGCAGAGGTCAAATAGAAGGTTAAGAAGTGCCCAGTGGATTTGGCAACCAGAGTCACTGGTAACCTTCAAAAGAACAATTTCAGTAGATTGTTAAGGGCAAAAGCCAGTTTACAGTAGGTTAAGGAGACAAAGGGAGATAAAAATTCCCAACATTCCATTCATTCATCTATCCCAGTGTGTACAGAGTGATTACTGTGTGTTTGTTGGCAG

Fig.S1


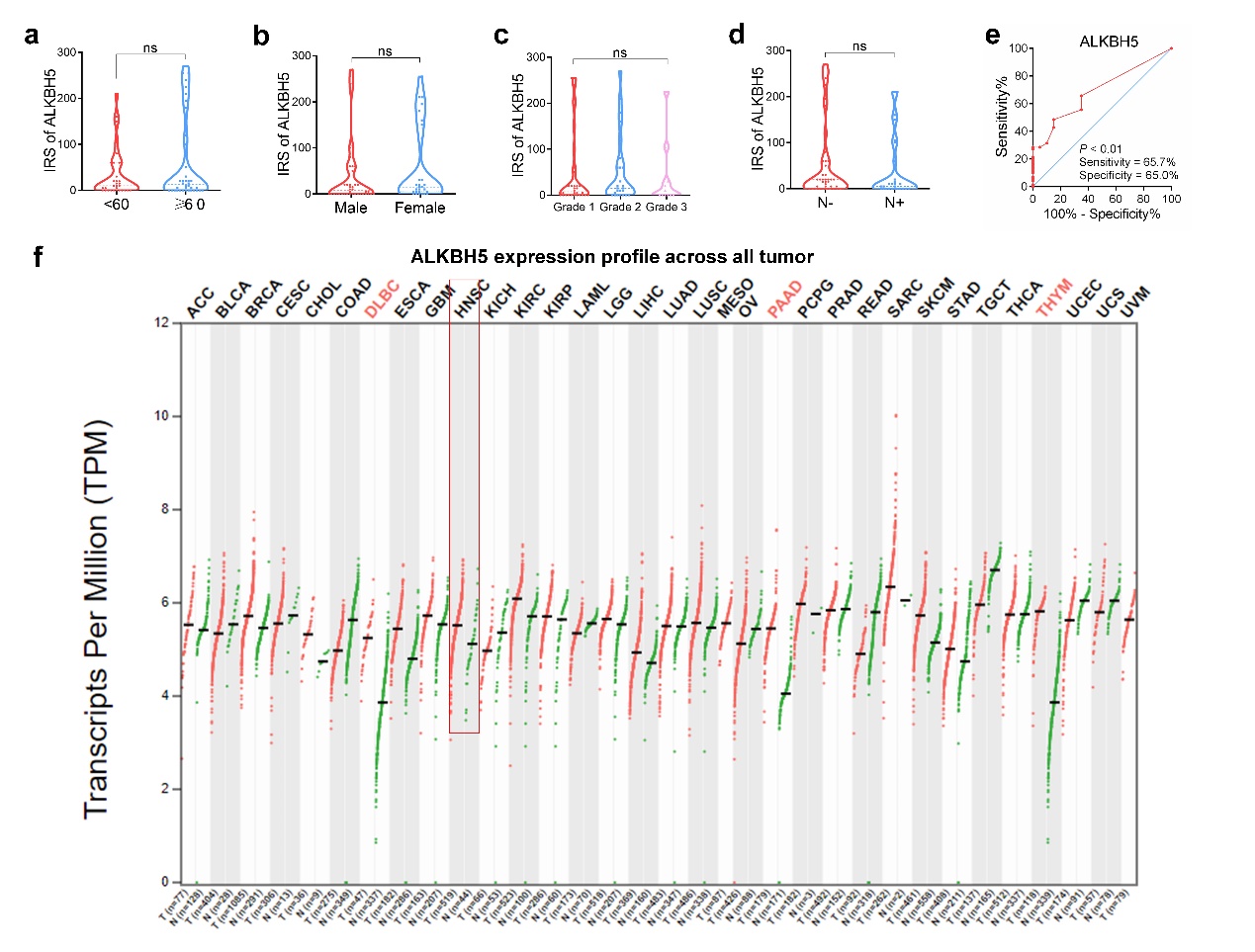


Fig.S2


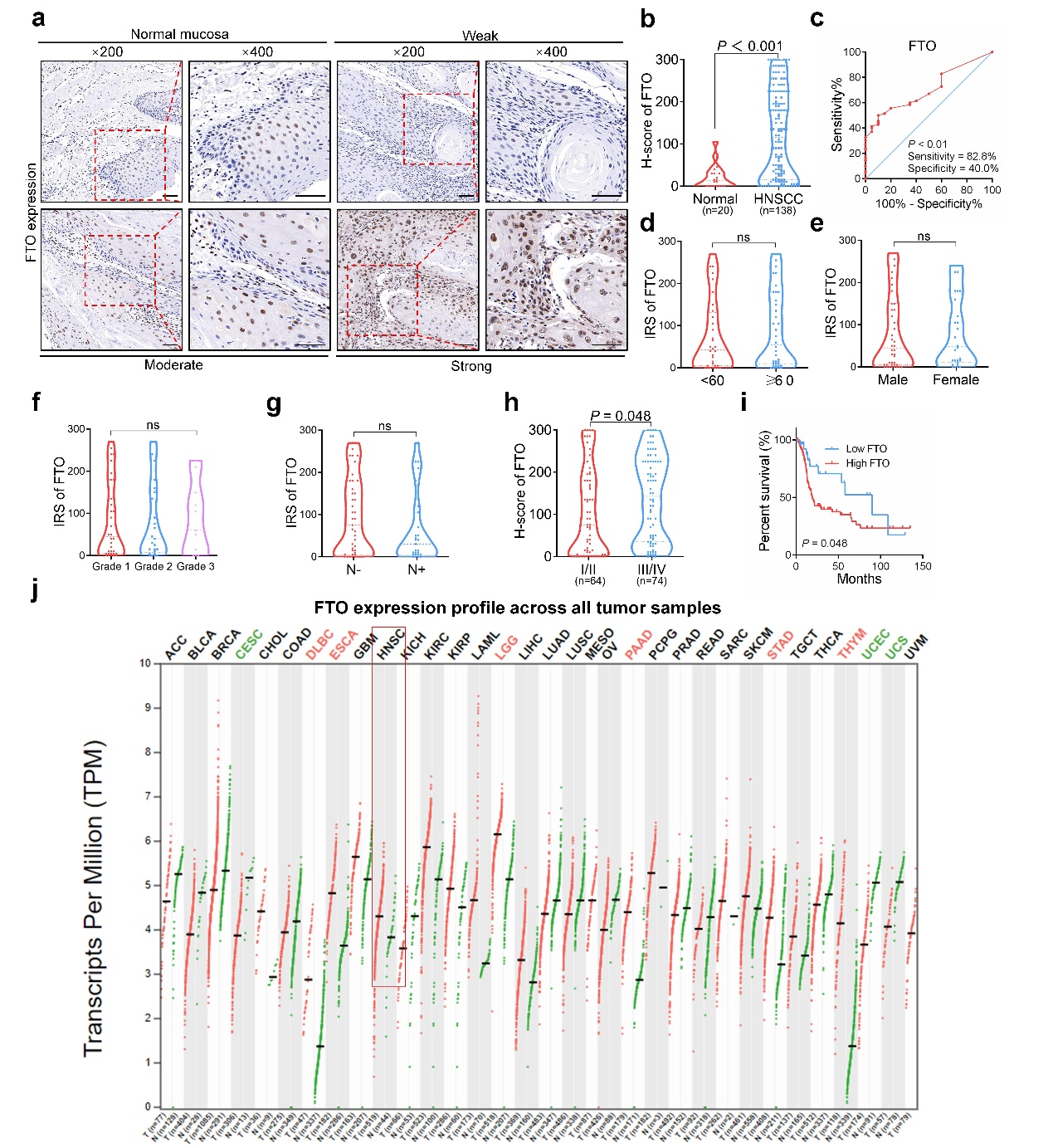


Fig.S3


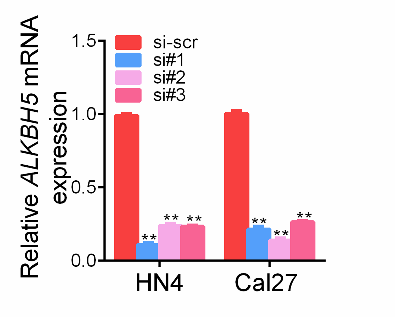


Fig.S4


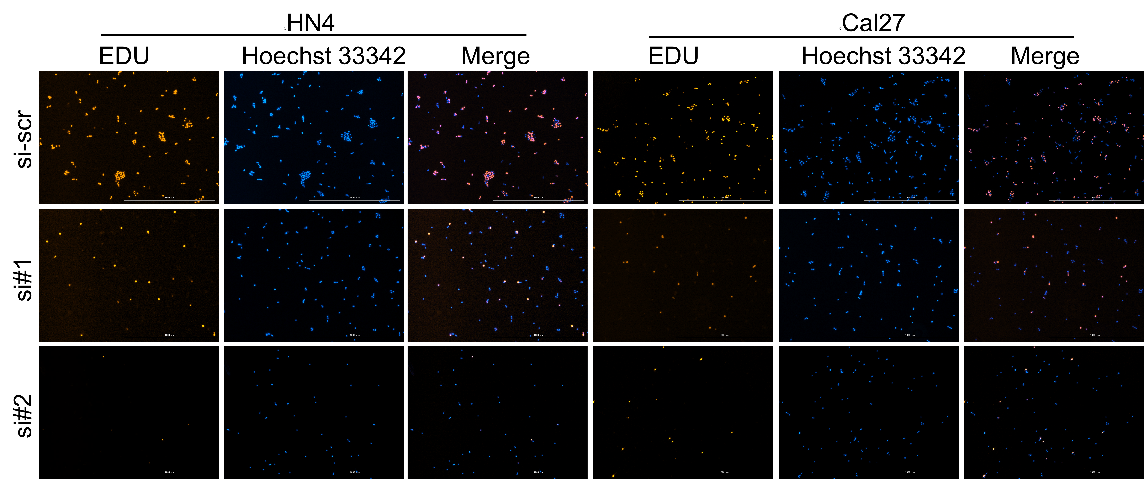


Fig.S5


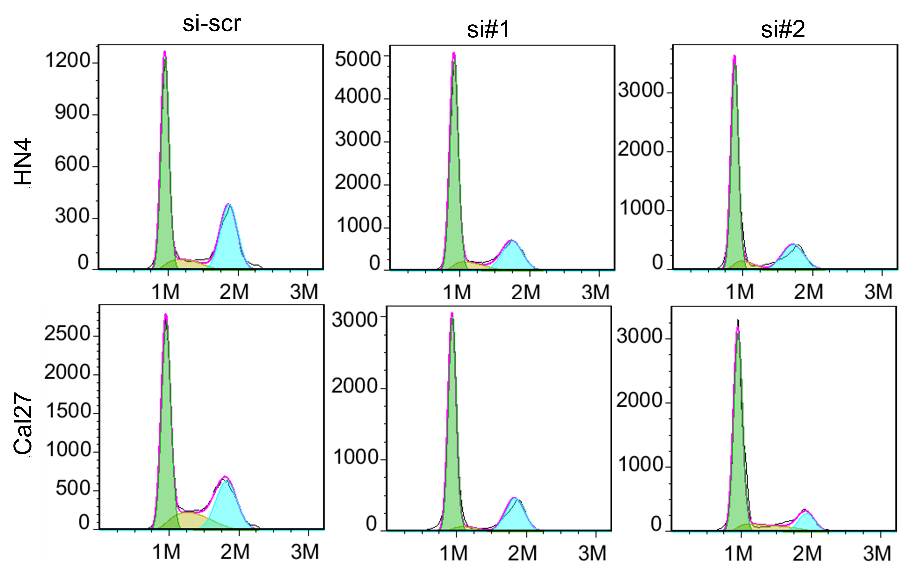


Fig.S6


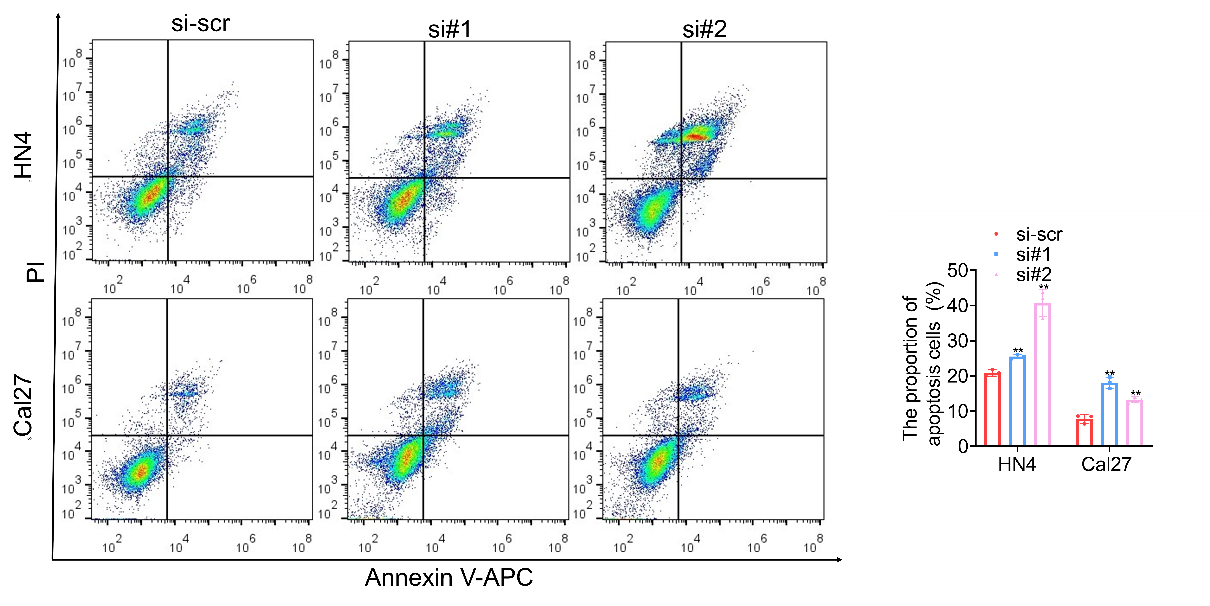


Fig.S7


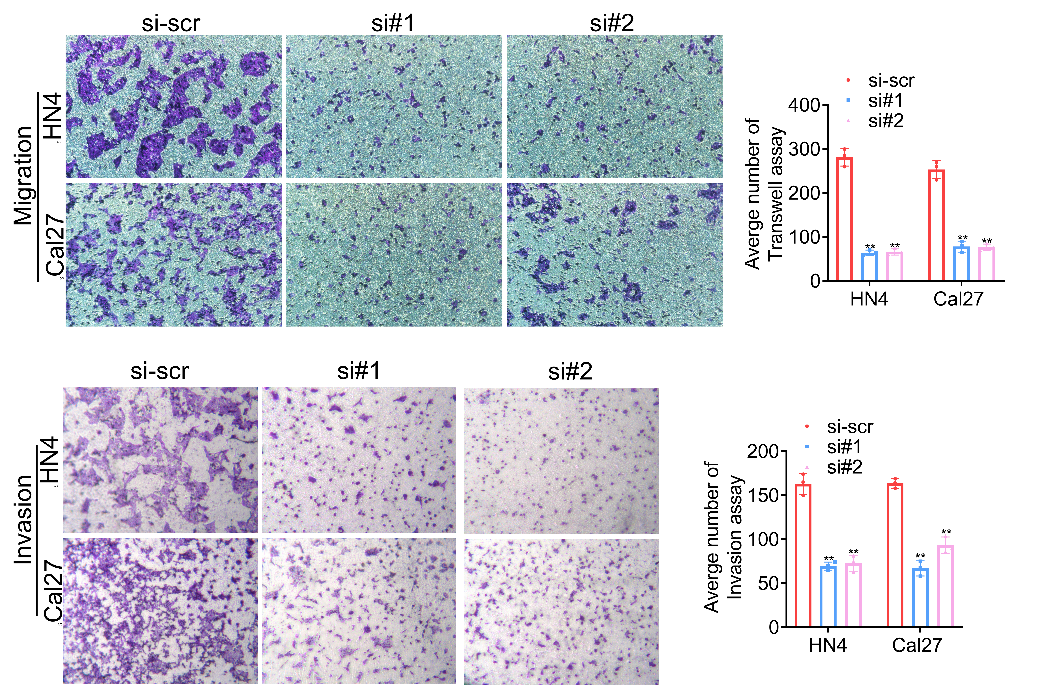


Fig.S8


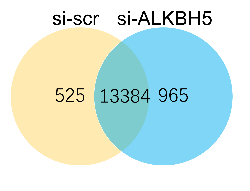


Fig. S9


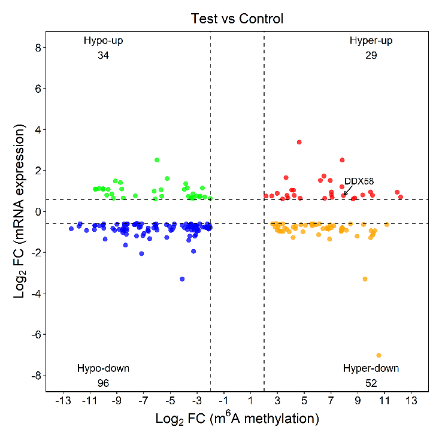


Fig.S10


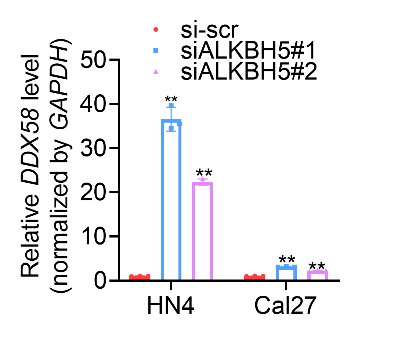


Fig. S11

| 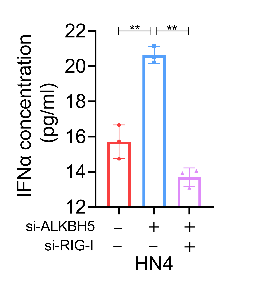 | 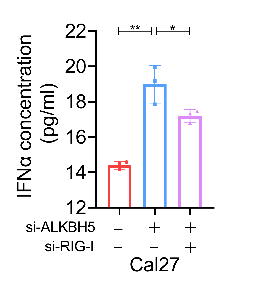 | 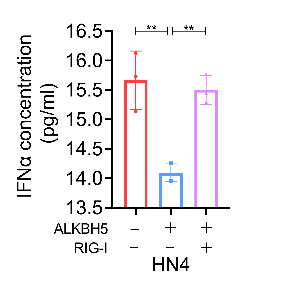 | 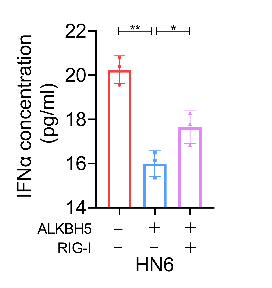 |
| --- | --- | --- | --- |

Fig.S12


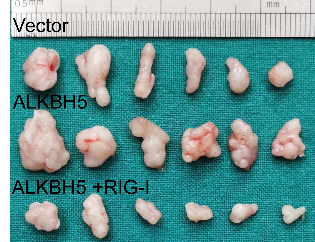


Fig.S13


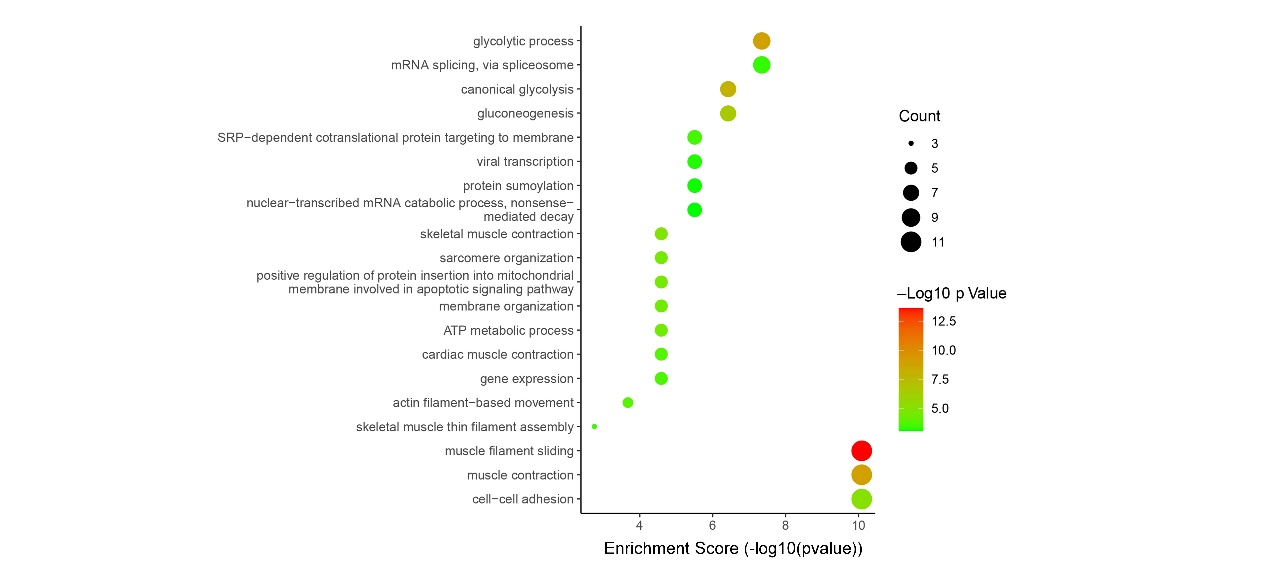


Fig.S14


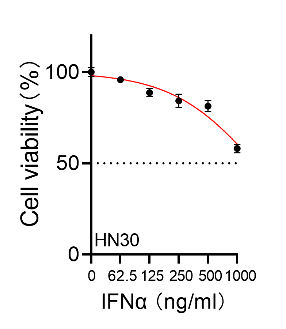

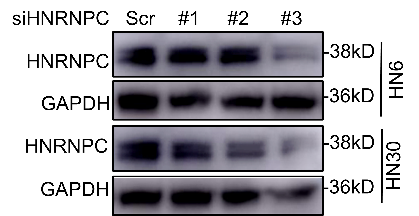


Fig.S15


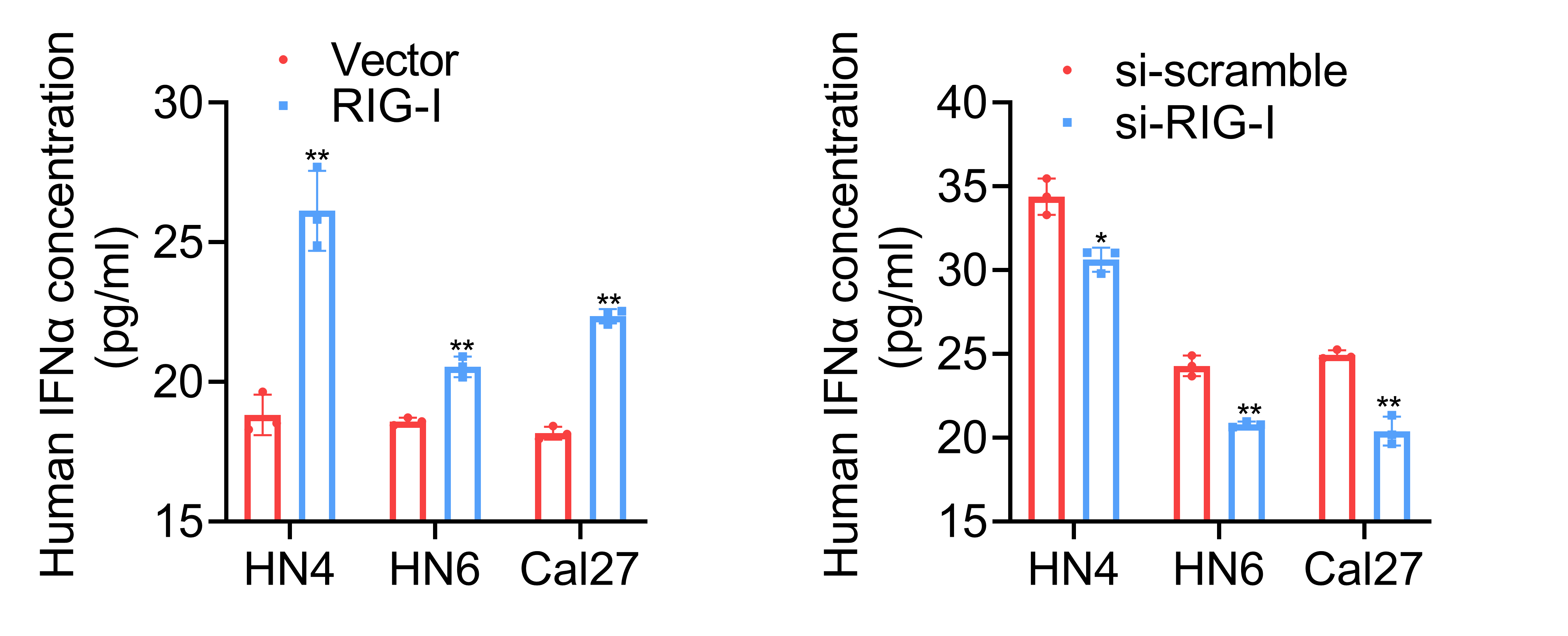


Fig.S16


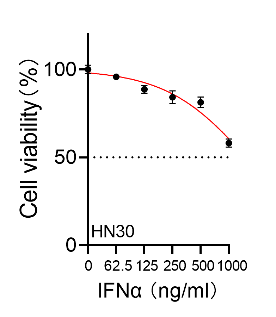

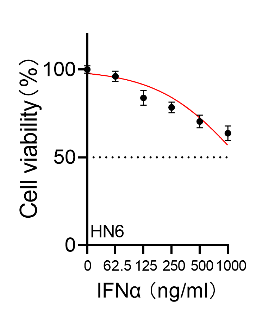

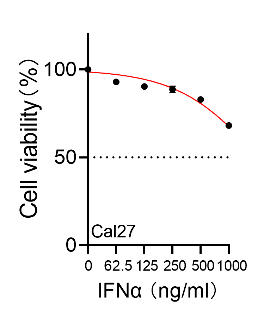

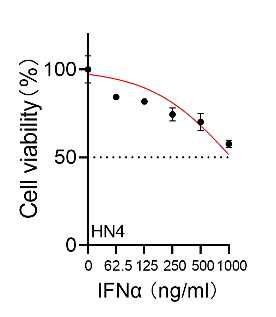


Fig.S17


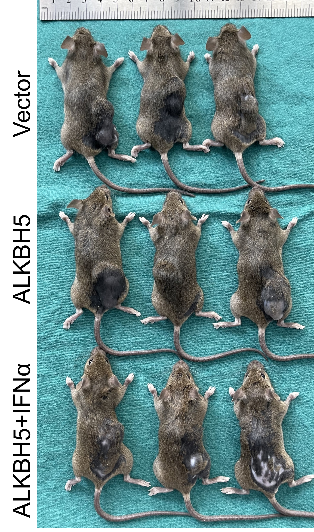


Fig.S18

| 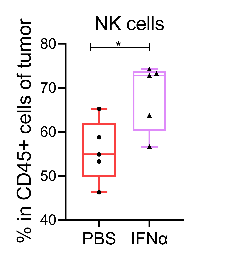 | 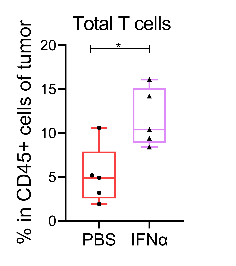 | 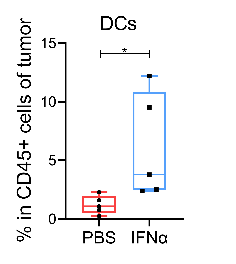 | 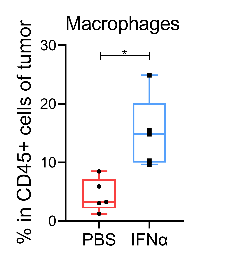 |
| --- | --- | --- | --- |

Fig.S19


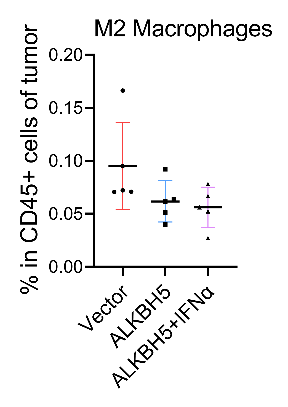

Supplement: Supplementary file 3 — Additional file 3: Supplementary Figure S1. The correlation between ALKBH5 expression and clinicopathological features in 138 HNSCC patients. (a-d) The correlation between the IRS of ALKBH5 and age, sex, pathological grade and lymph node status was analyzed in 138 HNSCC patients. (e) ROC curves were generated according to the IRS of ALKBH5 in HNSCC and normal controls. (f) The pancancer expression profile of ALKBH5 according to the GEPIA dataset. Figure S2. The correlation between FTO expression and clinicopathological characteristics in 138 HNSCC patients. (a) Representative images of immunohistochemical staining for FTO protein on a tissue microarray composed of 138 HNSCC tissues and 20 normal epithelial tissues. Scale bars: 100 μm. (b) The expression level of FTO was analyzed in HNSCC and the normal control. (c) ROC curves were generated according to the IRS of FTO in HNSCC and normal controls. (d-h) The correlation between the IRS of ALKBH5 and age, sex, pathological grade, lymph node status and TNM stage was analyzed in 138 HNSCC patients. (i) The Kaplan–Meier method with a two-tailed log-rank test was used to plot survival curves in The Cancer Genome Atlas (TCGA) HNSCC dataset with high and low FTO expression. The log-rank test was used to compare the survival rate. (j) The expression profile of FTO in pancancer tissues according to the GEPIA dataset. *P < 0.05, **P < 0.01. Figure S3. ALKBH5 mRNA was detected after siRNA transfection for 24 hours. Figure S4. The EdU assay was performed after siRNA transfection for 48 hours. Figure S5. The cell cycle distribution was analyzed using flow cytometry (PI staining) after transfection for 48 hours. Figure S6. Apoptotic cells were detected using flow cytometry (Annexin V/PI staining) after transfection for 48 hours. Figure S7. The migration and invasion capacities were detected using Transwell inserts with and without Matrigel, respectively, after transfection for 48 hours. Figure S8. Venn diagrams show differentially [file 12943_2022_1572_MOESM3_ESM.docx]
